# Tool for assessing food industry commitments and practices to address the double burden of malnutrition: a Delphi study

**DOI:** 10.1186/s12992-025-01175-8

**Published:** 2025-12-25

**Authors:** Carmen Klinger, Elochukwu C. Okanmelu, Peter Delobelle, Melissa A. Theurich, Daniela Rincón Camargo, Kurt Gedrich, Nicole Holliday, Eva A. Rehfuess, Olufunke Alaba, Zandile Mchiza, Estelle V. Lambert, Stefanie Vandevijvere, Lana Vanderlee, Gary Sacks, Peter von Philipsborn

**Affiliations:** 1 Institute for Medical Information Processing, Biometry and Epidemiology, Chair of Public Health and Health Services Research, Faculty of Medicine, LMU Munich, Elisabeth-Winterhalter-Weg 6, 81377 Munich, Germany; 2 Pettenkofer School of Public Health, Elisabeth-Winterhalter-Weg 6, 81377 Munich, Germany; 3 Chronic Disease Initiative for Africa, University of Cape Town, Groote Schuur Hospital, J47/86 Old Main Building, Observatory, Cape Town, 7925 South Africa; 4 Department of Public Health, Vrije Universiteit Brussel, Laarbeeklaan 101, Brussels, 1090 Belgium; 5 Research Group Public Health Nutrition, ZIEL – Institute for Food & Health, Technical University of Munich, Weihenstephaner Berg 1, 85354 Freising, Germany; 6 Health Economics Unit, School of Public Health, University of Cape Town, Barnard Fuller Building, Anzio Rd, Observatory, Cape Town, 7925 South Africa; 7 Non-Communicable Disease Research Unit, South African Medical Research Council, Francie van Zijl Drive, Parowvallei, Tygerberg, Cape Town, 7505 South Africa; 8 School of Public Health, University of the Western Cape, Robert Sobukwe Road, Bellville, Cape Town, 7535 South Africa; 9 Health through Physical Activity, Lifestyle, and Sport (HPALS) Research Centre, University of Cape Town, Boundary Road, Newlands, Cape Town, 7700 South Africa; 10 Department of Epidemiology and Public Health, Sciensano, Rue Juliette Wytsman 14, Brussels, 1050 Belgium; 11 École de Nutrition, Centre NUTRISS, Université Laval, 2440 boul. Hochelaga, Québec, G1V 0A6 Canada; 12 Global Centre for Preventive Health and Nutrition, Deakin University, 1 Gheringhap Street, Geelong, VIC 3220 Australia; 13 Chair of Public Health Nutrition, Faculty of Life Sciences, University of Bayreuth, Fritz-Hornschuch-Strasse 13, 95326 Kulmbach, Germany

**Keywords:** Undernutrition, Malnutrition, Food insecurity, Obesity, Noncommunicable diseases, Food industry, Commitments, Voluntary action, Expert consultation, Monitoring

## Abstract

**Background:**

Many low- and middle-income countries face a double burden of malnutrition, i.e., a co-occurrence of undernutrition with overweight, obesity, or other diet-related noncommunicable diseases. In an increasingly connected global food system, multinational and domestic food industry actors – through their commercial practices and corporate political activity – both contribute to the double burden of malnutrition and hold potential to address it. Systematic monitoring of relevant industry commitments and practices may help to hold industry accountable and foster constructive engagement. The *Business Impact Assessment - Obesity and population-level nutrition* (BIA-Obesity) tool has been developed to assess and benchmark food companies’ commitments and practices related to obesity and support for healthy diets at a national level.

**Methods:**

To enable the application of BIA-Obesity for countries facing a double burden of malnutrition, this study aimed to identify and select relevant best practice indicators for assessing food company commitments and practices regarding the double burden of malnutrition, with a focus on indicators not currently captured by the BIA-Obesity tool. A three-round Delphi study was conducted between April and October 2024, involving an international panel of experts.

**Results:**

From 52 invited experts, 30 contributed to our expert panel (response rate 58%). Based on a systematic review, 16 best practice indicators addressing the double burden of malnutrition were proposed. Consensus (i.e., group agreement of 75% or higher) for inclusion was reached for 8 indicators covering the production, distribution and marketing of (i) breastmilk substitutes and (ii) complementary foods, (iii) breastfeeding support and (iv) parental leave for employees, (v) food fortification, (vi) use of traditional foods, (vii) use of discounts and donations, and (viii) healthy diets at work. One additional indicator on corporate strategy was included as an overarching indicator.

**Conclusions:**

Food industry action may complement other efforts to address the double burden of malnutrition, such as public policies and investments. Tools like the extended BIA-Obesity framework can be used for a systematic monitoring of relevant industry commitments and practices and may help to disseminate and establish favourable industry practices as part of broader efforts to address the double burden of malnutrition in low- and middle-income countries.

**Clinical trial number:**

Not applicable.

**Supplementary Information:**

The online version contains supplementary material available at 10.1186/s12992-025-01175-8.

## Background

Many low- and middle-income countries (LMICs) face a double burden of malnutrition (DBM), defined as the co-occurrence of a relevant burden of undernutrition (wasting, stunting, underweight, and/or micronutrient deficiencies), alongside a relevant burden of overweight, obesity, or other diet-related noncommunicable diseases (NCDs) [1]. The DBM can be observed at the population level (community, municipality, local region, country, or world region), the household level, or at the individual level, at any given point in time or over the life course [1–3]. To emphasise the role of micronutrient deficiencies as a separate aspect, some organisations use the term ‘triple burden of malnutrition’ instead (e.g., the United Nations Children’s Fund (UNICEF) [4], the Food and Agriculture Organization of the United Nations (FAO) [5], and the International Food Policy Research Institute (IFPRI) [6]). According to a 2020 *Lancet* series, regions particularly affected by the DBM are Sub-Saharan Africa, South Asia, and East Asia & the Pacific, with roughly a third of all LMICs facing a substantial DBM [2]. Since the 1990s, the DBM has shifted from primarily affecting upper-middle-income economies to now predominantly impacting lower-middle-income and low-income economies, driven by a rise in overweight and obesity without a substantial decline in undernutrition in these countries [2].

The emergence of the DBM has coincided with, and is arguably partly driven by the nutrition transition, a population-level shift in dietary consumption (from traditional, high-cereal, high-fibre diets towards diets rich in refined sugar, fat and salt) and energy expenditure (due to changes in physical activity patterns towards a more sedentary lifestyle) [7, 8]. The nutrition transition occurs alongside broader societal shifts such as economic growth, urbanisation, aging populations, and transitions from infectious diseases towards NCDs. It is also influenced by globalisation leading to changes in food systems (defined as “the people, institutions, places, and activities that play a part in growing, processing, transporting, selling, marketing, and, ultimately, eating food” [9]) and food environments (defined as the physical, economic, political, and socio-cultural contexts influencing people’s decisions about acquiring, preparing, and consuming food [7, 10, 11].

Industry actors, such as domestic and multinational food and non-alcoholic beverage manufacturers, supermarkets, and quick-service restaurants, play an important role in shaping national food environments through both their commercial practices and corporate political activity. Their activities are primarily driven by the expectation to maximise shareholder value and generate profit through their business operations [12]. This includes activities in the fields of corporate strategy, product formulation, product labelling, product and brand promotion, product pricing, distribution and positioning, as well as external relationships and lobbying [13]. The latter includes strategies to shape public health policy, research and practice in ways that benefit food companies, such as hindering the development of adequate standards in the Codex Alimentarius Commission, or lobbying against the taxation of sugar-sweetened beverages and for self-regulation in areas such as marketing to children [14, 15]. By increasing their market penetration into LMICs over the past decades, globally operating multinational food companies have contributed to the globalisation of dietary patterns [2, 16]. Compared to high-income countries (HICs), government regulation of commercial activities is often less stringent in LMICs [17]. In case of existing regulations or standards, their implementation, monitoring and enforcement is frequently inadequate, e.g., in the case of marketing regulations of unhealthy foods and beverages towards children [18] or the composition of infant formula [19, 20], thus creating loopholes for unfavourable food industry practices [17].

Given the important role of industry practices in shaping population nutrition (i.e., the dietary quality and nutritional status of a population), several tools and frameworks have been developed to systematically assess and benchmark such practices and related commitments [21–23]. The Access to Nutrition initiative (ATNi), an international non-profit organisation, regularly publishes a global index evaluating and ranking the commitments, performance and disclosure practices of major global food and beverage manufacturers in addressing malnutrition in all its forms [24, 25]. ATNi has also conducted national assessments in countries such as India [26, 27] and the United States [28, 29]. In 2022, it released the first retailer index for the United Kingdom focused on obesity prevention [30, 31]. The World Benchmarking Alliance, a non-profit organisation representing over 300 members including businesses, financial institutions and non-governmental organisations, aims to measure company action in relation to the Sustainable Development Goals [22, 32]. Another tool to monitor the actions of the private food sector has been developed by the International Network for Food and Obesity/NCDs Research, Monitoring and Action Support (INFORMAS). INFORMAS is a global network of researchers and public health experts that monitors and benchmarks food environments and policies to increase healthy food environments and reduce obesity and NCDs [33]. Their *Business Impact Assessment - Obesity and population-level nutrition* (BIA-Obesity) tool is used to assess the voluntary commitments and practices of the most relevant food companies in a country (based on market share), in relation to obesity and population nutrition across six domains, benchmark them against international best practices and identify priority actions for change at company, sector and global level [13].

The methods of the BIA-Obesity tool are based on WHO recommendations, public health literature and the ATNi global index [13]. BIA-Obesity assessments have been conducted in Australia [34], Belgium [35, 36], Canada [37, 38], France [35], Ireland [39], Malaysia [40], New Zealand [41], and Thailand [42], and are currently being implemented in Portugal and Senegal [43]. BIA-Obesity does not explicitly address environmental sustainability, for which the complementary BIA-Sustainability tool has been developed by Mackay et al. (2022) [44]. This tool includes 35 indicators across 11 domains (e.g., packaging, emissions, food loss and waste) and has been applied in Australia [45] and Belgium [36]. While the BIA-Obesity tool allows for a comprehensive assessment of food industry commitments and practices regarding overweight, obesity and unhealthy diets, it does not systematically address issues related to the DBM, particularly concerning undernutrition (including stunting, wasting, underweight, and/or micronutrient deficiencies).

A systematic review [46], conducted prior to the present study, identified recommendations for the food industry to address undernutrition, as a single issue and within the context of a DBM. Building upon these findings, the present study aimed to extend BIA-Obesity, to develop a comprehensive and resource-efficient tool to assess food companies’ commitments and practices impacting population-level nutrition in contexts where a DBM exists. Specifically, the aim of the study was to identify best practice indicators regarding the DBM that are not currently captured by the BIA-Obesity tool, through a three-round Delphi process involving an international expert panel. The extension to the existing BIA-Obesity tool containing the new DBM indicators will be referred to as *Business Impact Assessment - Double Burden of Malnutrition* (BIA-DBM).

## Methods

### Overview

We conducted a Delphi study following the *Delphi studies in social and health sciences—Recommendations for an interdisciplinary standardized reporting* (DELPHISTAR) guidance [47]. Our study is based on an a priori protocol registered and published with the Open Science Framework [48]. In the following sections, we provide a summary of our methodology. Further details, including the DELPHISTAR checklist, are provided in the supplementary material.

### Delphi methodology

We applied a Delphi technique, first described by Dalkey & Helmer in 1963 [49], and subsequently applied to various research and practice contexts [50]. The basic principle is that feedback is obtained from an expert panel in several rounds through questionnaires or interviews on an issue with limited or inconsistent evidence [47, 49]. The aggregated group perspective is shared with participants after each survey round and panellists can reassess their evaluations, taking into account the additional information provided [50]. This method of controlled interaction among respondents purposely avoids the drawbacks associated with traditional expert engagement methods like round-table discussions, and encourages independent thought among experts, facilitating the gradual formation of well-considered opinions [51, 52]. Over the past few years, several variations of the Delphi methodology have been developed. In this study, we followed the Group Delphi technique by integrating a workshop between two anonymous online surveys [47]. This approach was chosen to allow experts to provide contextual justification for any divergent judgment. It has also been shown to present a benefit for participants, as the workshop provides an opportunity to exchange ideas and network with colleagues in related fields [52–54].

### Recruitment of an expert panel

The composition of the expert panel, in terms of breadth and depth of expertise, is crucial for a successful Delphi study. In order to decide on the experts to be invited, we considered the following aspects formulated by Beiderbeck et al. (2021).: (i) size of expert panel (i.e., 15–40 experts in each Delphi round), (ii) level of expertise (i.e., person working in or with research focus on the topic), (iii) level of heterogeneity (i.e., representation from both HICs and LMICs, and from various sectors), (iv) level of interest (i.e., personal interest in the study results), and (v) access to members of the panel (i.e., focus on mid-level professionals and established contacts) [50]. Detailed information on the selection process and composition of the expert panel is provided in the supplementary material. In order to participate in the study, participants were also required to disclose any potential conflict of interest (i.e., any interest that may affect, or may reasonably be perceived to affect, the expert’s objectivity and independence related to the scope of the research). Participation in the study was contingent on the declared potential conflicts of interest, which were individually reviewed by the study team.

### Study design

**Fig. 1:**
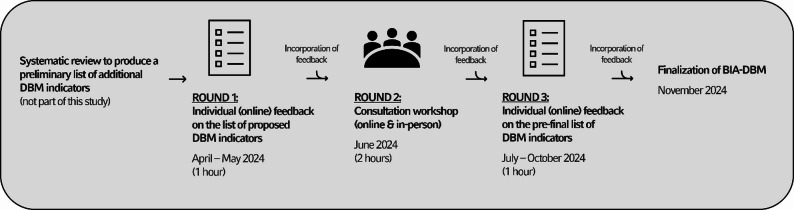
Delphi process to select indicators for assessing food industry action in countries with a DBM. BIA = Business Impact Assessment, DBM = Double Burden of Malnutrition

The Delphi study was conducted by an interdisciplinary team with representatives from the fields of nutrition and dietetics, public health, epidemiology, health economics, health policy, and medicine. The study was conducted between April and October 2024, with two rounds of anonymous online feedback and a 2-hour collaborative workshop (online or in-person) (see Fig. 1). Participants did not have to take part in the workshop and could choose to participate in either one or both online surveys. The total number of Delphi rounds (online surveys and workshop) was defined in advance to be a maximum of three.

### Initial review of the existing BIA-obesity tool and preselection of new indicators

This study is based on our systematic review [46], which aimed to comprehensively identify, map, and synthesise recommendations, lived experiences, and existing frameworks regarding the role of the food industry in addressing undernutrition, as a single issue and within the context of a DBM, in LMICs. We synthesised our findings into 28 overarching recommendations for the food industry – in the context of this work defined as the organised, regulated and structured sector of the food system operating within established legal and economic frameworks and encompassing a wide range of actors, including food and beverage manufacturers, retailers and supermarkets, quick-service restaurants, wholesalers, and caterers. We also provide additional sector-specific recommendations for food and non-alcoholic beverage manufacturers (i.e., companies producing packaged food and drink products), retailers (i.e., companies selling goods to the public in relatively small quantities for use or consumption rather than for resale), and quick-service restaurants (i.e., chain restaurants – often also referred to as fast food outlets – including chain restaurants that offer sit-down casual dining). Most of our findings aligned with the existing BIA-Obesity domains, such as recommendations on product (re-)formulation, nutrition labelling, marketing practices, the availability, accessibility and affordability of healthy foods, and multi-stakeholder processes. However, we also identified two additional relevant domains: supply chain and workforce. In view of the results of the systematic review, we examined the BIA-Obesity tool to identify any DBM aspects already covered by existing indicators. Additionally, we considered the following four criteria for the preselection of new indicators, as used by the INFORMAS team in a similar process^1^:

- Focus on food environments: exclusion of non-food environment actions such as broader food system action, women’s empowerment, financial and social protection, aid programmes;
- Feasibility: only select key DBM indicators (those most often referred to in the literature) as the original BIA-Obesity tool already has a high number of indicators;
- Compatibility: alignment with the original BIA-Obesity tool to ensure comparability of assessments across countries and over time;
- Relevance: indicator acts as a key leverage (according to literature) to improve population nutrition.

To refine the formulation of the proposed novel indicators, we organised multiple sessions within the author team. We then assessed the comprehensibility of the new indicators by consulting academic colleagues not otherwise involved in the project.

### Data collection and analysis

The two online surveys were conducted via SoSci Survey [55]. The first online survey was conducted between April and May 2024, and the second online survey between July and October 2024. Both survey setups were pre-tested by co-authors and further colleagues prior to distribution. The 2-hour workshop was offered either virtually via Zoom (3 June 2024) or in-person (12 June 2024) at the World Public Health Nutrition Congress in London, England. Feedback received during the workshops was anonymised prior to data analysis. All statistical analyses were undertaken using Microsoft Excel version 2410 and RStudio version 4.2.3. We did not conduct any group-specific analysis or weighting of participant feedback. The qualitative feedback provided was thematically summarised by one author (DR) and checked by a second author (CK).

#### Round 1: individual online feedback

We introduced participants to the original BIA-Obesity tool and invited them to assess the pre-selected new DBM indicators on a 5-point Likert scale ranging from 1 (very low) to 5 (very high) according to their (i) relevance (i.e., contribution to addressing the DBM), (ii) achievability (i.e., likelihood of adoption and feasibility of implementation), and (iii) measurability (i.e., availability of data recorded in a standardised manner). In addition, we gave participants the opportunity to provide comments and/or suggestions on the formulation of each indicator and to propose new indicators [51]. We also requested information about the participants’ profession and their self-assessed years of relevant experience in the field [50, 56].

The three assessment criteria (relevance, achievability, and measurability) were analysed descriptively and presented as histograms [57]. At this stage, no indicators were excluded, as we assumed that perceptions might evolve as the indicators were refined throughout the Delphi process. The histograms picturing the participants’ own assessments in comparison to the group’s assessment were fed back to each individual prior to the next Delphi round (i.e., the workshop) [51]. The qualitative feedback regarding each indicator and the BIA-Obesity tool overall was collected and thematically summarised in a text file and informed the (re)phrasing of indicators [58]. This text file was also shared with the participants, indicating how we had addressed the various suggestions.

#### Round 2: consultation workshop

During the workshop, we first presented the scope of the BIA-Obesity tool and the aims of the adaptation process. We also clarified any overarching questions and comments that arose during the first Delphi round. We then presented the revised list of proposed DBM indicators, discussed the indicators’ assessment (relevance, achievability, and measurability), and collected additional suggestions and comments on how to improve them. Both workshops were recorded using Zoom. We collected and thematically summarised the feedback received during both workshops in a text file and prepared a pre-final list of indicators [58].

#### Round 3: individual online feedback

We provided participants with the revised list of indicators and a summary of the main discussion points for each indicator. Participants were asked to assess each indicator for inclusion in the adaptation of the BIA-Obesity tool on a 7-point Likert scale ranging from 1 (strongly in favour of excluding the indicator) to 7 (strongly in favour of including the indicator). For this final selection of indicators, we opted for a more detailed rating scale (7-point instead of 5-point Likert scale), ensuring greater precision in this crucial step. Participants were also invited to provide any final suggestions regarding each indicator and/or the overall tool. We also asked them to evaluate the Delphi process based on four items (compilation of expert panel; instructions and feedback; new insights; overall outcome) on a 5-point Likert scale ranging from 1 (not at all satisfied) to 5 (extremely satisfied), or 1 (strongly disagree) to 5 (strongly agree).

The rating on the inclusion of indicators was analysed descriptively and presented in the form of a histogram [57]. In line with standard Delphi processes [59, 60], we defined consensus as a group agreement of 75% or higher in the second online survey round (includes Likert scale ratings from 5 – ‘somewhat in favour of inclusion’ to 7 – ‘strongly in favour of inclusion’). The qualitative feedback regarding each indicator and the BIA-Obesity tool overall was collected in a text file and informed the final (re)phrasing of indicators. The evaluation of the entire Delphi process was analysed descriptively.

### Finalisation of BIA-DBM

Based on the final rating, we selected the indicators to be included in the new BIA-DBM extension. In the case of an indicator rating being very close to but not meeting or exceeding the 75% consensus threshold, the research team reserved the right to discuss these cases internally and together with the INFORMAS private sector module leaders [49]. We conducted final adaptations, including the scoring of new indicators, in close coordination with INFORMAS.

## Results

### Characteristics of the expert panel

We invited 52 individuals to participate in our Delphi expert panel. In total, 30 experts (response rate 58%) agreed to contribute to the panel, and participated in at least one of the three Delphi rounds. Most participants (80%, *n* = 24) identified themselves as researchers. Additionally, the panel included three government officials, three civil society representatives, two dietitians, and one United Nations officer. Notably, three participants fell into multiple of these categories (see Figure s1, supplementary material). Participants’ self-reported amount of relevant experience in the field varied substantially. Among them, 24% had 1–5 years of experience, 28% had 6–10 years, 20% reported 11–15 years, and 20% had 16–20 years of relevant experience, respectively. In addition, two participants reported having over 25 years of experience in the field (see Figure s2).

### Participation in the Delphi process

Twenty-six experts (87% response rate) participated in the first Delphi round, 23 (77% response rate) attended the consultation workshop, and 19 (63% response rate) completed the third and final Delphi round (see Table 1).

**Table 1:** Number of participating experts over the Delphi process, according to current profession(s)

	Round 1 (online survey)	Round 2 (workshop)		Round 3 (online survey)
		online	in-person	
Researcher / university staff	21	7	11	15
Civil society representative	3	2	-	2
Government official	2	3	-	2
United Nations officer	1	1	-	1
Dietitian	1	1	1	1
**Total number of experts**	**26**	**23**		**19**

### Initial review of the existing BIA-Obesity tool and preselection of new indicators

Some indicators within the original BIA-Obesity tool already address aspects relevant to the DBM (e.g., reduction of nutrients of concern in products; provision of front-of-pack nutrient labelling; restrictions on the targeted marketing of unhealthy foods to children). After reviewing the existing BIA-Obesity tool [13] and the findings of our systematic review on this topic [46], we drafted a preliminary list of 16 best practice indicators addressing the DBM, currently not being part of BIA-Obesity (see Tables s2-s3). The rationale for excluding certain indicators previously identified in our systematic review from consideration in this Delphi study is documented in Table s1.

### Round 1: individual online feedback

During the first Delphi round, participants assessed the relevance of 14 (out of 16) indicators as ‘fair’ to ‘very high’. For 12 (out of 16) indicators the achievability was rated as ‘fair’ to ‘very high’. Participants rated the measurability of half of the proposed 16 indicators as ‘fair’ to ‘very high’. Figure s3 presents the histograms for each indicator assessment, which were shared with the expert panel after the first Delphi round.

Overall, participants highlighted various overarching issues in their qualitative comments:

- Several indicators should be further disaggregated for a better understanding and to facilitate the indicators’ measurement and monitoring (e.g., see Table s3 – indicators DBM-PARENT and DBM-BREAST);
- Ambiguous or undefined terminology (e.g., ‘healthy’, ‘adequate’, ‘complementary foods’, etc.) can lead to intentional or unintentional manipulation by the food industry and should be defined or avoided;
- Some indicators were deemed to lack a relevant, population-wide impact in addressing the DBM;
- The nutritional quality of foods, as well as food safety aspects and cultural acceptability and desirability should be considered in the formulation of the new indicators; and
- For some indicators, the applicability to a specific food industry sector was questioned (e.g., the likelihood that a quick-service restaurant enables its employees to maintain a healthy and affordable diet during working hours).

A detailed summary of the qualitative feedback received for each indicator and how we addressed it is provided in Table s4. Any modifications regarding the indicators’ wording based on the first Delphi round are noted in Table s3.

### Round 2: consultation workshop

During the expert consultation workshop, we focused on those indicators where the greatest need for further clarification and discussion had been identified. In addition to the qualitative feedback received during the first Delphi round, the following main themes emerged during the workshops:

- The potential misuse of indicators by the food industry for marketing, corporate social responsibility or corporate political activity purposes;
- Underlying political food system issues (e.g., food fortification as a medium-, not a long-term strategy);
- The potential of company culture change and innovation (i.e., transformation from within the company, leading by example); and
- The consideration of unintended consequences (e.g., increase of fat and/or sugar content in company reformulation strategies, disruption of local markets in communities).

A summary of all points of discussion during the two consultation workshops and how we addressed them is presented in Table s5. Any modifications regarding the indicators’ wording based on the second Delphi round can be found in Table s3.

### Round 3: individual online feedback

**Fig. 2:**
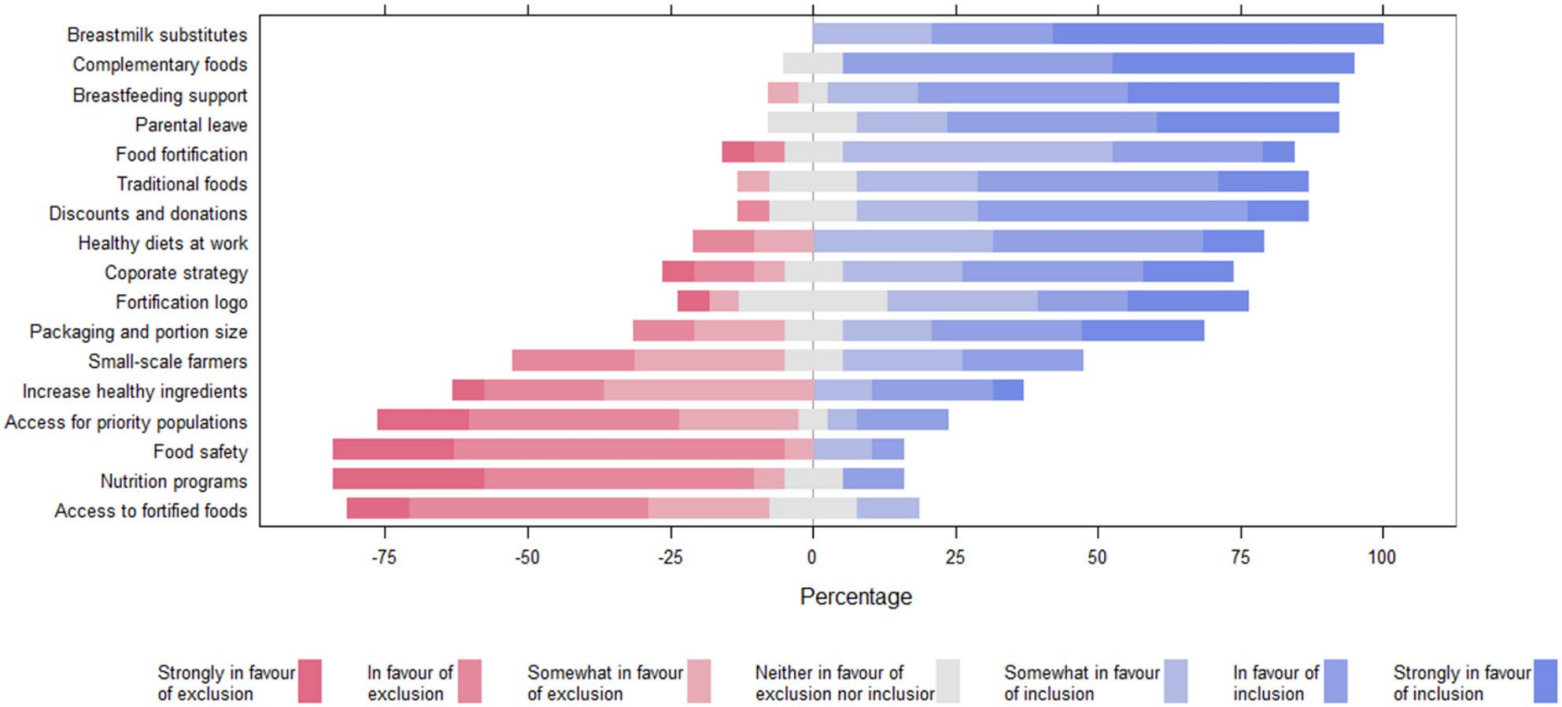
Final rating regarding the inclusion of proposed double burden of malnutrition indicators

Figure 2 shows the final rating regarding the inclusion of proposed indicators during the third Delphi round. Consensus for inclusion was reached for eight indicators: *Breastmilk substitutes* (DBM-CODE; expert consensus: 100%), *Complementary foods* (DBM-COMPL; expert consensus: 89%), *Breastfeeding support* (DBM-BREAST; expert consensus: 89%), *Parental leave* (DBM-PARENT; expert consensus: 84%), *Food fortification* (DBM-FORTI; expert consensus: 79%), *Traditional foods* (DBM-TRAD; expert consensus: 79%), *Discounts and donations* (DBM-DONAT; expert consensus: 79%), and *Healthy diets at work* (DBM-WORK; expert consensus: 79%). All final indicator ratings are presented in Table s6.

A summary of the qualitative feedback received during the third Delphi round and how we addressed it is presented in Table s7. We identified the following overarching issues:

- Difficulties regarding the measurement of indicators;
- Proposed reduced weighting of workplace indicators or the removal of these indicators for country contexts where the workforce contributing to food manufacturing and/or retailing is relatively small; and
- Applicability aspects due to the multinational context and different organisational structures within companies (e.g., regarding the increased use of traditional foods in food production or with regard to parental leave policies).

Experts also proposed further minor refinements to the indicators’ wording. Any final modifications based on the third Delphi round can be found in Table s3.

### Finalisation of BIA-DBM

In addition to the eight indicators that reached expert consensus for inclusion, the author team selected *Corporate strategy* (DBM-STRAT; expert consensus: 68%) as an additional indicator for the BIA-DBM extension. This decision was made to complement an existing BIA-Obesity indicator (STRAT2 in the original BIA-Obesity tool [13]).

**Fig. 3:**
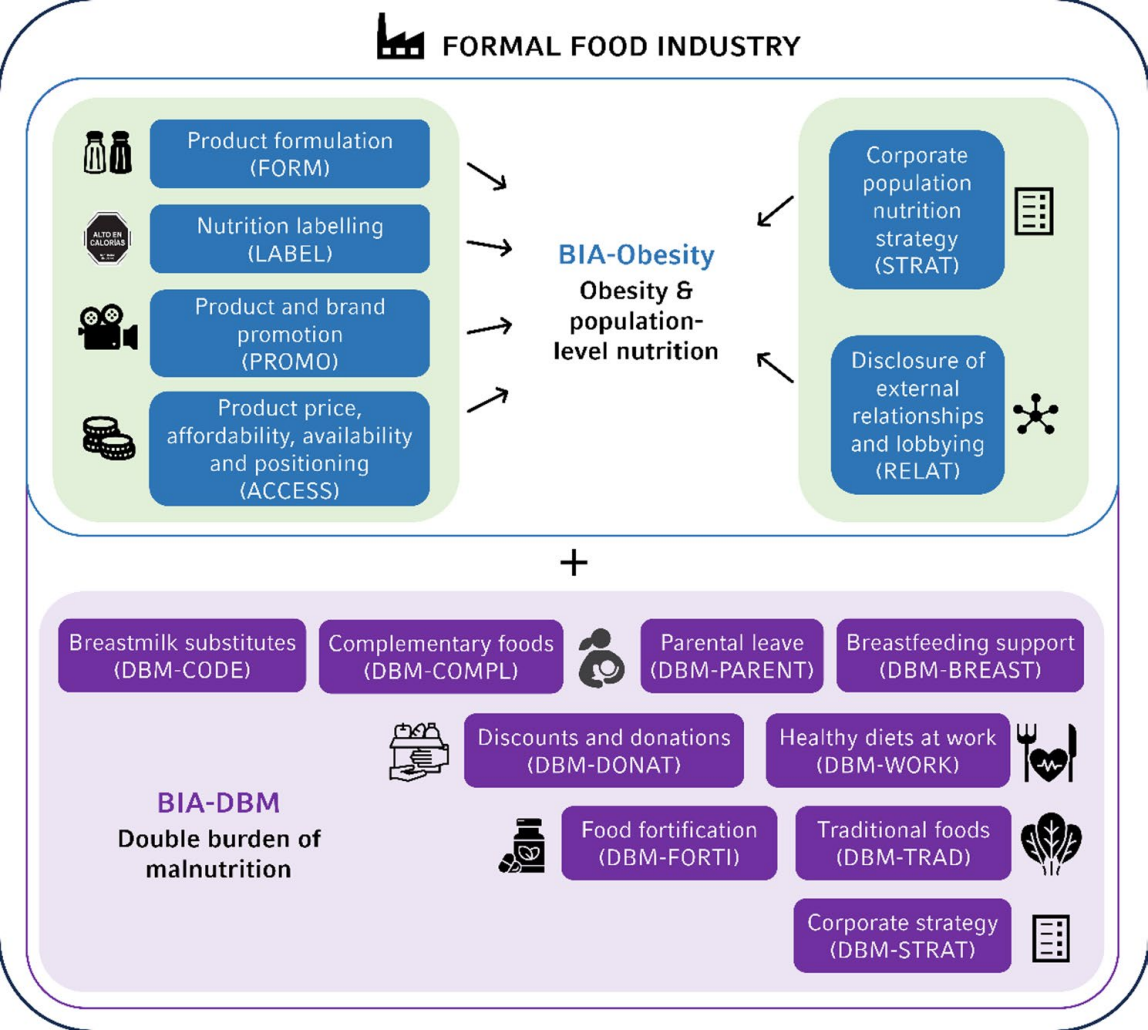
BIA-Obesity tool and BIA-DBM extension for countries with a double burden of malnutrition (own illustration). BIA = Business Impact Assessment, DBM = Double Burden of Malnutrition

The new DBM indicators can be integrated into the existing BIA-Obesity tool as part of the tool’s adaptation to the respective country context, or presented as a separate, complementary BIA-DBM score (see Fig. 3). Workplace indicators, i.e., *Parental leave* (DBM-PARENT), *Breastfeeding support* (DBM-BREAST), *Healthy diets at work* (DBM-WORK), may be excluded in country contexts where the proportion of the national workforce employed in the three food industry sectors is comparatively low, as their relevance to national-level assessments may be diminished in such contexts.

The scoring scheme for the new BIA-DBM indicators was developed together with the INFORMAS private sector group. Similar to the original BIA-Obesity tool, focus is put on (i) the existence of any commitment/policy, (ii) the public disclosure of such commitment/policy, (iii) the regular reporting of practice/performance in this area, including in relation to targets/commitments, and (iv) a national (country-specific) focus of the commitment/policy (see Table 2). As for BIA-Obesity, this scoring scheme is flexible, allowing implementers to make adaptations based on the national context.

**Table 2:** Business impact assessment – double burden of malnutrition (BIA-DBM) indicators

BIA-DBM ID	Short description	Detailed description	Scoring	Max points
DBM-STRAT *Sectors: M*,* S*,* R*	Corporate strategy	Does the company’s commitment to improving population nutrition and health (where it exists) specifically mention addressing undernutrition (wasting, stunting, underweight, micronutrient deficiencies), linked to national health and development priorities?	5: Yes, commitment specifically refers to addressing undernutrition (wasting, stunting, underweight, micronutrient deficiencies), as aligned with or as outlined in key government policy documents 2.5 Yes, commitment refers to addressing undernutrition (wasting, stunting, underweight, micronutrient deficiencies), through their philanthropic activities 0: No, commitment does not refer to undernutrition, as aligned with or as outlined in key government policy documents N/A if there is no commitment to population nutrition and health	5
DBM-FORTI *Sectors: M*,* S*	Food fortification	For companies producing/using/distributing fortified or enriched foods^1^, does the company commit to and/or report on complying with applicable national, regional, or (if not available) international standards or recommendations (e.g., guidance limiting fortification to nutritious foods^2^, staples and condiments)?	Select all that apply (max 10 points): 2.5: Existence of any commitment/policy 2.5: Public disclosure of the commitment/policy 2.5: Regular reporting of practice/performance in this area, including in relation to targets/commitments 2.5: National (country-specific) focus of the commitment/policy	10
DBM-TRAD *Sectors: M*,* S*,* R*	Traditional foods	Does the company commit to and/or report on increasing the use of traditional foods^3^, that are culturally acceptable and nutritious (e.g., sweet potato, amaranth, millets) in the production of nutritious foods^2^, in line with national or international dietary guidelines?	Select all that apply (max 10 points): 2: Existence of any commitment/policy 2: Limit use to the production of nutritious foods^2^ only 2: Alignment with national or international dietary guidelines 2: Public disclosure of the commitment/policy 2: Regular reporting of practice/performance in this area, including in relation to targets/commitments	10*
DBM-CODE *Sectors: M*,* S*	Breastmilk substitutes	Does the company commit to and/or report on complying with ‘The International Code of Marketing of Breastmilk Substitutes’ and all subsequent related WHA resolutions, as well as relevant Codex Alimentarius standards (e.g., CXS 72-1981)?	Select all that apply (max 10 points): 2: Existence of any commitment/policy to comply with ‘The Code’ and subsequent related WHA resolutions 2: Existence of any commitment/policy to comply with relevant Codex Alimentarius standards (e.g., CXS 72-1981) 2: Public disclosure of the commitment/policy 2: Regular reporting of practice/performance in this area, including in relation to targets/commitments 2: National (country-specific) focus of the commitment/policy	10
DBM-COMPL *Sectors: M*,* S*	Complementary foods	Does the company commit to and/or report on producing, marketing, labelling, and distributing commercially available complementary foods (CACFs)^5^ only in line with national evidence-informed recommendations, or (if not available) international guidance (e.g., the International Code of Marketing of Breastmilk Substitutes, Codex Alimentarius (CXS 156–1987, CAC/GL 8-1991, CXS 73-1981, CXS 74-1981), or WHO NPPM)?	Select all that apply (max 10 points): 1: Existence of any commitment/policy on the *production* of CACFs in line with national or international guidance 1: Existence of any commitment/policy on the *marketing* of CACFs in line with national or international guidance 1: Existence of any commitment/policy on the *labelling* of CACFs in line with national or international guidance 1: Existence of any commitment/policy on the *distribution* of CACFs in line with national or international guidance 2: Public disclosure of the commitment/policy 2: Regular reporting of practice/performance in this area, including in relation to targets/commitments 2: National (country-specific) focus of the commitment/policy	10
DBM-DONAT *Sectors: M*,* S*,* R*	Discounts and donations	Does the company commit to and/or report on applying discounts on nutritious food^2^ that is considered safe (i.e., within ‘use by date’ and unspoiled) but that cannot be sold at the regular price, and/or donating it to people in need?	Select all that apply (max 10 points): 2.5: Existence of any commitment/policy 2.5: Public disclosure of the commitment/policy 2.5: Regular reporting of practice/performance in this area, including in relation to targets/commitments 2.5: National (country-specific) focus of the commitment/policy	10**
DBM-PARENT *Sectors: M*,* S*,* R*	Parental leave	Does the company commit to and/or report on providing primary caregivers^6^ at least 14 weeks of paid parental leave?	Select all that apply (max 5 points): 1: Existence of any commitment to provide at least 14 weeks of paid maternity leave 1: Existence of any commitment/policy to provide monetary top-ups to governmental legislation 1: Existence of any commitment/policy ensuring paid parental leave for fathers/partners 1: Public disclosure of the commitment/policy 1: National (country-specific) focus of the commitment/policy	5
DBM-BREAST *Sectors: M*,* S*,* R*	Breastfeeding support	Does the company commit to and/or report on providing breastfeeding mothers with appropriate working arrangements (e.g., paid nursing breaks) and facilities at work (e.g., to breastfeed, express and store breastmilk), in line with national, regional or international guidance?	Select all that apply (max 5 points): 1: Existence of any commitment/policy on appropriate working arrangements 1: Existence of any commitment/policy on appropriate facilities at work 1: Alignment with national, regional or international guidance 1: Public disclosure of the commitment/policy 1: National (country-specific) focus of the commitment/policy	5
DBM-WORK *Sectors: M*,* S*,* R*	Healthy diets at work	Does the company commit to and/or report on having measures in place to ensure that employees can practice a healthy diet^7^ at an affordable price during working hours (e.g., company cafeterias serving nutritious foods^2^, food vouchers which can be used for nutritious foods^2^)?	Select all that apply (max 5 points): 1: Existence of any commitment/policy 1: Particular reference to affordability 1: Public disclosure of the commitment/policy 1: Regular reporting of practice/performance in this area, including in relation to targets/commitments 1: National (country-specific) focus of the commitment/policy	5
BIA-DBM SCORE				70

^1^Fortified or enriched foods: The addition of micronutrients (vitamins and minerals) to foods to enhance their nutritional value and provide health benefits, to restore nutrients lost during processing, and to prevent micronutrient deficiencies in the general population and specific groups like children, pregnant women, or social programme beneficiaries [61].

^2^Nutritious foods: Safe foods that contribute essential nutrients such as vitamins and minerals (micronutrients), fibre and other components to healthy diets that are beneficial for growth, health and development, guarding against malnutrition. In healthy foods, the presence of nutrients of public health concern is minimised [62].

^3^Traditional foods: Local foods (such as leafy vegetables, berries, small animals, or any other traditional food source from local biodiversity) that have long been an important part of people’s diets, and which are usually nutritious, affordable and adapted to local growing conditions and cultural traditions [63].

^4^Culturally appropriate foods: “the qualification of particular foods as appropriate to eat, in a particular manner, in a particular context. It is a relational phenomenon, arising through interaction between the embodied, enculturated dispositions of the eater(s) themselves and the socio-material context of consumption (including socially-shared standards of normal or suitable conduct, available foodstuffs, and other social actors” [64].

^5^Commercially available complementary foods (CACFs): “All foods and beverage products that are specifically marketed as suitable for feeding infants and young children up to 36 months of age and that are complementary to breast milk or breast-milk substitutes” [65].

^6^Primary caregiver: The main individual responsible for the care and upbringing of an infant / a child (usually parents, spouses, but could also be legal guardians).

^7^Healthy diet: Diets that are in line with applicable national, regional or (if not available) international food-based dietary guidelines [62]. Typically, healthy diets are characterised as adequate (providing enough essential nutrients to prevent deficiencies and promote health, without excess), balanced (in energy intake, and energy sources (i.e., fats, carbohydrates, proteins)), moderate (in consumption of foods, nutrients or other compounds associated with detrimental health effects), and diverse (including a variety of nutritious foods within and across food groups to favour nutrient adequacy and consumption of other health promoting substances) [66].

*If the company refers only to ‘local’ foods, 2 points are still awarded for the presence of a commitment or policy.

**If the company does not specify whether this applies to nutritious products, the awarded points are halved.

BIA = Business Impact Assessment, DBM = Double Burden of Malnutrition, M = Food and non-alcoholic beverage manufacturers, S = Supermarkets, R = Quick-service restaurants, WHO NPPM = WHO Nutrient and promotion profile model

Indicators presented in this study which did not reach consensus for inclusion may still be used in countries where researchers deem them highly relevant (e.g., access of small-scale farmers to companies’ supply chains). In such cases, we recommend reviewing the discussion points in Tables s4, s5 and s7, and adhering to the general scoring scheme outlined above.

### Evaluation of the Delphi process

Overall, participants in the Delphi process (*n* = 19, response rate: 63%) were very satisfied with the compilation of the expert panel (e.g., size of panel, area(s) of expertise, heterogeneity) reflected in a median rating of 4 out of 5 (IQR 4–5). Experts were also very satisfied with the overall outcome of the Delphi process (i.e., the proposed indicators in the final survey round), with this item receiving a median rating of 4 out of 5 (IQR 4–5). Regarding the instructions and feedback provided (e.g., study invitation, online survey instructions, feedback of individual assessment vs. group assessment, summary of main discussion points), experts were extremely satisfied, with a median rating of 5 out of 5 (IQR 4–5). Additionally, they strongly agreed that participating in the Delphi process provided them with new insights (median rating of 5 out of 5, IQR 4–5).

## Discussion

### Summary and interpretation of results

As part of this Delphi study, we identified nine indicators to assess food industry commitments and practices addressing the DBM. For countries experiencing a DBM, the proposed BIA-DBM indicators can be used to complement the existing BIA-Obesity tool, which primarily focuses on addressing and preventing obesity and unhealthy diets. Among the nine new indicators, four specifically address infant and young child feeding: *Breastmilk substitutes* (DBM-CODE), *Complementary foods* (DBM-COMPL), *Parental leave* (DBM-PARENT), and *Breastfeeding support* (DBM-BREAST). These indicators focus on the first 1,000 days of life (roughly from conception to the age of two years), a crucial period where nutrition and care determine a child’s survival, physical and mental development, and future opportunities [67, 68]. The food industry can play an important role in supporting exclusive breastfeeding for the first six months of life, as well as the introduction of nutritionally adequate complementary foods with continued breastfeeding, by taking concerted actions. These include offering paid parental leave for primary caregivers, providing appropriate working arrangements and facilities for breastfeeding, and by following the International Code of Marketing of Breastmilk Substitutes [20], subsequent relevant WHA resolutions [69], and Codex Alimentarius standards for breastmilk substitutes and commercially available complementary foods [70–74]. The relevance of appropriate infant and young child feeding practices in addressing undernutrition, overweight and obesity is supported by various studies and international organisations [75–80] and was also a key aspect considered in extending the INFORMAS *Healthy Food Environment Policy Index* (Food-EPI) tool for contexts characterised by a DBM^2^.

Two new indicators refer to product formulation: *Food fortification* (DBM-FORTI) and *Traditional foods* (DBM-TRAD). In the public health community, food fortification (e.g., with vitamin A, iron, iodine, zinc, folic acid) has long been promoted as a cost-effective strategy with wide population reach to combat micronutrient deficiencies and the concomitant severe health issues in LMICs [61, 81, 82]. Substantial advancements have been achieved in several countries through large-scale food fortification of staple foods – notably, iodized salt and flour fortified with a range of micronutrients, which have helped to reduce anaemia, goitre, and neural tube defects, among others [83]. However, in recent years, criticism of food fortification has grown, with concerns that it is only a short- to medium-term solution that fails to address the structural causes of malnutrition, such as poverty, food insecurity, poor dietary diversity, and inadequate healthcare access [82, 84] – concerns echoed by some of our participating experts. Moreover, the benefits of food fortification can be compromised by weak monitoring and quality control, which can result in inconsistent nutrient levels in fortified products [82, 85]. Additionally, there are concerns that fortification is used as a marketing tool, when micronutrients are added to products of poor nutritional value to create a misleading impression of healthfulness [86]. Therefore, our expert panel emphasised that, in principle, food companies are not encouraged to enrich or fortify foods where this is not mandated or recommended by public health bodies. Food fortification and enrichment should always be done in compliance with applicable national, regional, or global standards and recommendations. This may include guidance on limiting fortification to healthy foods, as well as staples and condiments. The increased use of traditional foods (e.g., sweet potato, amaranth, millets) in the production of nutritious foods is in line with recommendations by WHO and FAO which highlight the relevance of traditional and indigenous crops as part of a balanced and sustainable diet [87]. It has been argued that by being drought-resistant and nutrient-dense, many traditional crops have the potential to enhance food security in LMICs [63].

The new indicator *Discounts and donations* (DBM-DONAT) aims to encourage companies to apply discounts on nutritious food that is considered safe but cannot be sold at the regular price (e.g., due to exceeding the best-before date), and/or donating these products to people in need. Noting that donations and discounts of breastmilk substitutes are not permitted by ‘The International Code of Marketing of Breast-Milk Substitutes’ and subsequent related WHA resolutions [20, 69]. There has also been criticism that using surplus food to address food insecurity can deflect from addressing the structural causes of food insecurity and may also reduce companies’ motivation to prevent food loss and waste in the first place [88–90]. While surplus food is often safe for consumption, improper handling, storage, or distribution can lead to contamination or spoilage, potentially causing foodborne illnesses [88, 91].

The indicator *Healthy diets at work* (DBM-WORK) assesses whether companies implement measures that enable employees to practice a healthy diet at an affordable price during working hours. This includes initiatives such as company cafeterias serving nutritious foods or food vouchers which can be used for nutritious options. In many LMICs, a substantial share of the workforce is employed in the food sector, highlighting the indicator’s potential for population-level impact. In addition, our expert panel emphasised the role of workplace environments in leading by example and fostering culture change from within food companies. Recent studies have demonstrated that comprehensive workforce nutrition programmes (i.e., various interventions offered to employees through workplace delivery structures, including access to nutritious food), especially those targeting high-risk employees, can be effective in improving nutrition and health outcomes [92–94]. However, these findings are largely based on evidence from HICs, and there remains a research gap regarding their effectiveness in LMIC contexts. The indicator may be less applicable in countries where the proportion of the national workforce employed in the food industry is relatively low.

One last indicator, *Corporate strategy* (DBM-STRAT), evaluates if a company’s corporate strategy specifically mentions addressing undernutrition. This indicator was additionally included to complement an existing BIA-Obesity indicator (STRAT2) focusing on obesity and NCDs prevention as part of a company’s corporate strategy.

Through the BIA-DBM extension, together with existing BIA-Obesity indicators in the domains of corporate population nutrition strategy (STRAT), product formulation (FORM), nutrition labelling (LABEL), product and brand promotion (PROMO), and product price, affordability, availability and positioning (ACCESS), the assessment considers key dimensions of the human right to food, such as availability, accessibility, and adequacy [95, 96].

### Strengths and limitations of the study

Our study has several strengths. We conducted this study in accordance with current methodological best practice recommendations as expressed by the DELPHISTAR guidance [47]. By applying a Delphi technique, we ensured the consideration of diverse opinions across multiple continents on the issue of voluntary commitments and practices by the food industry. Additionally, we refrained from including food industry representatives to avoid any conflicts of interest. This may, however, affect the practical applicability of the indicators. Although expert participation declined across the three Delphi rounds, this primarily concerned the larger subgroup of researchers and university staff, and the final round still captured various perspectives from research, civil society, government, and international organisations. The 16 indicators initially proposed in this study were based on a previously conducted systematic review [46], ensuring that the Delphi study was informed by the latest available external evidence. The workshops (online and face-to-face) offered ample room for discussion, thereby maximising expert participation in the second round of the Delphi study. The results of the process evaluation of the Delphi study indicate high levels of satisfaction with the compilation of the expert panel, the instructions provided, the overall study outcomes and the insights generated. This in turn supports the validity and relevance of the new indicators.

Our study also has a number of limitations. We did not collect information on the participants’ sex, gender, and ethnicity. This limits our understanding of representation with regard to these aspects and potential biases and may also affect the generalisability of the study results. Given the technical nature of the indicators and the global application of our approach, we did not include end consumers in our expert panel. Instead, we involved civil society representatives. Although all participants of the final online survey round (*n* = 19) took part in the process evaluation, the survey was solely pseudo-anonymous (i.e., researchers CK and PD have access to the identification key that link the survey and workshop contributions to the experts’ identities), which may have introduced desirability bias. We considered only indicators not yet covered by the existing BIA-Obesity tool and therefore did not include indicators on topics such as marketing of foods high in fat, salt and/or sugar. We also did not consider indicators on corporate political activity, as established frameworks focused on this issue exist [97, 98]. Finally, it must be noted that both BIA-Obesity and BIA-DBM focus on commitments and reporting practices and may not assess the entire extent to which commitments are implemented in practice. This is particularly important, as voluntary commitments do not necessarily translate into comprehensive and effective action and are a known strategy by the food industry to delay the adoption of mandatory regulation [98, 99].

#### Implications for practice and future research

The WHO, among others, has clearly identified the practices of the food industry as a major contributor to the global rise in overweight, obesity and NCDs [100, 101]. Thow et al. (2024) showed that in Ghana and South Africa, two countries facing a substantial DBM, the food industry primarily viewed its role as ensuring food sufficiency, with limited attention to nutritional quality [102]. Providing industry actors with appropriate indicators may help shift this perception and promote recognition of their responsibility in shaping healthy food environments. Besides, applying BIA-DBM together with BIA-Obesity can contribute to the independent monitoring of, and generate key insights into food industry action in LMICs. This in turn, helps to hold food industry actors accountable for their contributions to healthier, sustainable and equitable food environments [103]. To gain a comprehensive understanding of national food industry activity, these assessments should be carried out repeatedly and should be accompanied by observational studies of actual implementation (e.g., in supermarkets and quick-service restaurants) [104–106], as well as in-depth analyses of the industry’s corporate political activity [97, 98]. In Australia, the implementation of the BIA-Obesity tool has increased companies’ awareness of their role in addressing population nutrition and has influenced their nutrition-related commitments and reporting practices [107]. A number of caveats do, however, apply. Food industry action on addressing the DBM should be seen as complementary to, and not as a substitute for government action. Engaging with the food industry through tools such as BIA-Obesity and BIA-DBM may carry the risk of unduly emphasising its role in combating all forms of malnutrition over its role in contributing to the DBM, and obesity in particular. Furthermore, there is a risk that companies at the top of the ranking may use their placement for brand-promotion purposes, despite low absolute scores and limited corresponding action in practice – a criticism raised by some observers in the context of the ATNi assessments [108]. While the formal food industry is expanding in many LMICs, low-income populations often rely on informal markets for food procurement, such as small shops and street vendors. Interventions and effective monitoring in the informal food sector are therefore essential to complement the findings of this study and ensure equitable access to healthy foods. Next steps will involve pilot-testing the new BIA-DBM extension in countries experiencing different levels of the DBM, including South Africa [109]. Future research should also further explore if and how independent assessments of food industry’s commitments and practices ultimately impact industry activities and public policy.

## Conclusions

BIA-DBM builds on the existing BIA-Obesity tool by providing a comprehensive and resource-efficient tool for assessing national food industry commitments and practices in countries with high rates of undernutrition, overweight, obesity, and diet-related NCDs. Implementation of BIA-DBM may contribute to strengthening corporate accountability, identifying best practices, and enhancing capacity for implementation and monitoring. To effectively address the DBM, a comprehensive societal approach is needed. This includes respective action from the food industry, as well as strong regulatory frameworks accompanied by effective implementation, sustained public investments, and other forms of government action.

## Supplementary Information

Below is the link to the electronic supplementary material.

Supplementary Material 1

## Data Availability

The datasets generated and/or analysed during the current study are not publicly available to avoid possible identification of the experts involved but are available from the corresponding author on reasonable request.

## Footnotes

1 Personal communications with Stefanie Vandevijvere.

2 Personal communications with Stefanie Vandevijvere.

## Abbreviations

ATNi: Access to Nutrition initiative
BIA-Obesity: Business Impact Assessment – Obesity and population-level nutrition
BIA-DBM: Business Impact Assessment – Double Burden of Malnutrition
CACFs: Commercially available complementary foods
DBM: Double burden of malnutrition
DELPHISTAR: Delphi studies in social and health sciences-Recommendations for an interdisciplinary standardized reporting
FAO: Food and Agriculture Organization of the United Nations
Food-EPI: Healthy Food Environment Policy Index
HICs: High-income countries
INFORMAS: International Network for Food and Obesity/NCDs Research Monitoring and Action Support
IQR: Interquartile range
LMICs: Low- and middle-income countries
NCDs: Noncommunicable diseases
The Code: The International Code of Marketing of Breast-milk Substitutes
UNICEF: United Nations Children’s Fund
WHA: World Health Assembly
WHO: World Health Organization
WHO NPPM: WHO Nutrient and promotion profile model
