# Supplementary material for: Tool for assessing food industry commitments and practices to address the double burden of malnutrition: a Delphi study
Source: Global Health. 2025 Dec 25;22:14. doi: 10.1186/s12992-025-01175-8 (PMC12849613; doi:10.1186/s12992-025-01175-8)
Supplement: Supplementary file 1 — Supplementary Material 1 [file 12992_2025_1175_MOESM1_ESM.pdf]

Supplementary material

Supplementary material to: Carmen Klinger, Elochukwu C Okanmelu, Peter Delobelle, Melissa A Theurich, Daniela Rincon Camargo, Kurt Gedrich, Nicole Holliday, Eva A Rehfuess, Olufunke Alaba, Zandile Mchiza, Estelle V Lambert, Stefanie Vandevijvere, Lana Vanderlee, Gary Sacks, Peter von Philipsborn (2025): *Tool for Assessing Food Industry Commitments and Practices to Address the Double Burden of Malnutrition: A Delphi Study.* Manuscript submitted to Globalization & Health, November 2025

Corresponding author: Carmen Klinger, Institute for Medical Information Processing, Biometry and Epidemiology; Chair of Public Health and Health Services Research; Faculty of Medicine; LMU Munich, Elisabeth-Winterhalter-Weg 6, 81377 Munich, Germany; cklinger@ibe.med.uni-muenchen.de

Table of content

1. Detailed description of the methodology ... 2
   1.1. Recruitment of an expert panel ... 2
      1.1.1. Selection criteria ... 2
      1.1.2. Composition of the expert panel ... 3
   1.2. Rationale for not pursuing certain indicators for discussion in the Delphi study ... 4
   1.3. Information about method consulting ... 8
   1.4. Indicator codes used during the conduction of the Delphi study ... 9
2. Code used in the statistical analyses ... 9
   2.1. R code for individual indicator histograms (first Delphi round) ... 9
   2.2. R code for final rating histogram (third Delphi round) ... 9
3. Additional results ... 11
   3.1. First Delphi round: assessment of relevance, achievability and measurability ... 15
   3.2. First Delphi round: qualitative feedback ... 17
   3.3. Second Delphi round: qualitative feedback ... 28
   3.4. Third Delphi round: final rating regarding the inclusion of indicators ... 34
   3.5. Third Delphi round: qualitative feedback ... 34
4. Differences between protocol and manuscript ... 42
5. Reflexivity statement ... 43
6. DELPHISTAR guidance ... 44
7. References ... 48

1. Detailed description of the methodology

1.1. Recruitment of an expert panel

In order to decide on the experts to be invited to our Delphi study, we created a preliminary list of individuals from academia, civil society organisations, government, and United Nations agencies with expertise in private food industry commitments and/or the commercial determinants of health – defined as 'the systems, practices, and pathways through which commercial actors drive health and equity' (1) – with a particular focus on the DBM. This list was based on i) individuals being authors of included documents in our systematic review on the topic (2), ii) individuals' affiliation and involvement with existing assessment tools (e.g., BIA-Obesity, ATNi), as well as iii) our own professional networks. Furthermore, for the selection of relevant experts, we considered the following aspects formulated by Beiderbeck *et al.* (2021): i) size of expert panel, ii) level of expertise, iii) level of heterogeneity, iv) level of interest, v) access to members of the panel (3). Experts were contacted via email and invited to the panel. Follow-up reminder emails were sent by the author group as needed. Along with the study invitation, experts received a detailed information sheet outlining the study's objectives, the expected scope of their contribution, details on the processing, analysis, and storage of the data, as well as potential risks and benefits associated with participation.

1.1.1. Selection criteria

Size of expert panel

We aimed to include at least 15 and up to 40 experts in each step of our Delphi study. Existing guidance on Delphi studies recommends a condensed group of experts for specialized topics, as research shows that the variety in additional qualitative comments generally decreases when the number of experts reaches 30-40 (3, 4). Given the broad scope of the topic, we aimed to strike a balance between the number of experts included and the level of heterogeneity, without reaching the point of diminishing returns.

Level of expertise

We considered any person an expert who is working in or whose research focuses on one of the original BIA-Obesity key domains (corporate population nutrition strategy; product formulation; product labelling; product and brand promotion; product accessibility; relationships with external organizations) (5), as well as additional relevant domains identified through a systematic review (2). These cover but are not limited to aspects of food safety, infant and young child feeding, and food fortification. Relevant experience was determined by scientific research interest (including PhD students) in the respective area.

Level of heterogeneity

Research indicates that a lack of diversity (usually referring to demographic characteristics as well as professional experience) in a Delphi panel can introduce bias (6). Therefore, we sought to achieve representation from both high-income and low- and middle-income countries, and we included experts from academia, non-governmental organizations, and civil society organizations, as well as policymakers. We refrained from including food industry representatives in the methodological development of the framework to avoid any real or perceived conflicts of interest.

Level of interest

An expert's personal interest in the survey results might facilitate their investment and participation (3). We compiled an initial list of potential experts in such a way that everyone listed presumably has a medium to high interest in the study results. Prior to the initiation of the consultations, we circulated conflict of interest forms to all persons who agreed to participate in our survey. Three experts indicated that they regularly engage with food industry representatives as part of their research (e.g., during data collection), however they have not accepted any money/funding from food industry or related entities, and the food industry was/is not involved in the research design, results or reporting. No other potential conflicts of interest were reported.

Access to the panel

We ranked our initial list of experts based on the level of accessibility and years of experience. The decision was to primarily reach out to mid-level professionals and/or persons we have already had established contacts with, in order to increase the likelihood of participation. In the case of a lack of response from, or interest in taking part in the study by a proposed expert, we contacted experts from our preliminary expert list with a similar background to fill their spots.

1.1.2. Composition of the expert panel

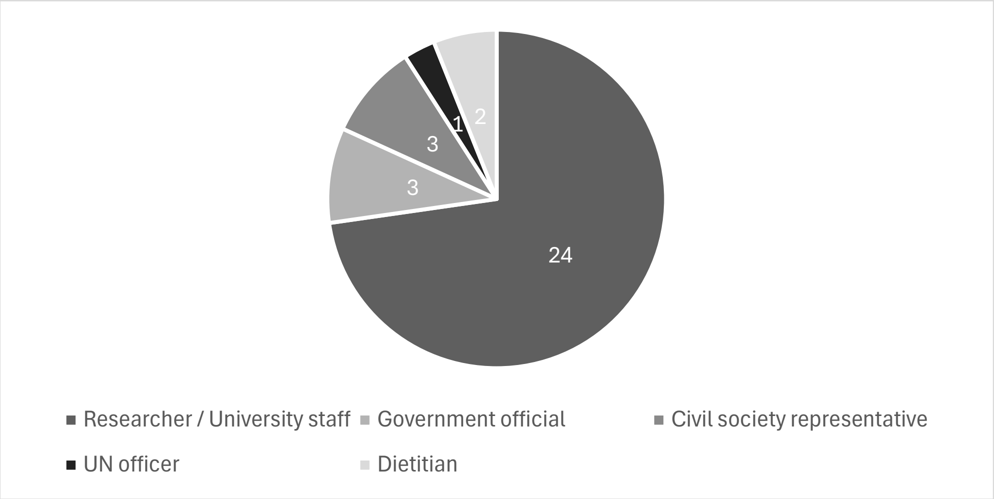

Figure s1: Current profession of participating experts; multiple options per person possible (n=30)

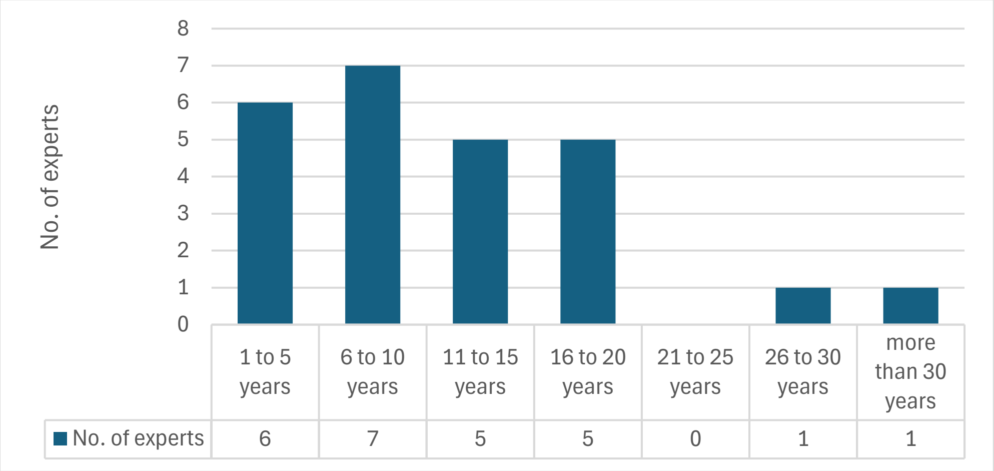

Figure s2: Experts' self-assessed years of relevant experience in the field of public health nutrition / commercial determinants of health / private food sector industry's policies and practices (n=25)

1.2. Rationale for not pursuing certain indicators for discussion in the Delphi study

Table s1: Rationale for pursuing / not pursuing certain indicators for discussion in the Delphi study, based on the results of a systematic review (2); the indicators initially proposed during the first Delphi round may differ from those in bold in the list below, as both research efforts were conducted simultaneously

Action category	Recommendation for the Food Industry (in general)	Inclusion/Exclusion in Delphi study (with reason)
**Corporate strategy**	Companies should make nutrition a long-term, board-level priority, e.g., by committing to relevant SDGs (such as SDG 2, 3, and/or 12), and/or to other global goals such as those of the Tokyo Nutrition for Growth (N4G) Pledge	Exclusion: Already covered by BIA-Obesity
**Product formulation**	Companies should reduce levels of nutrients of concern (salt/sodium, sugar, and saturated fat) in relevant products[1]	Exclusion: Already covered by BIA-Obesity
	Companies should eliminate industrially produced trans-fat in relevant products[1]	Exclusion: Already covered by BIA-Obesity
	Companies should reduce calories in relevant products[1]	Exclusion: Already covered by BIA-Obesity
	**Companies should increase the content of fruits, vegetables, nuts, legumes and/or wholegrains in relevant products**	**Inclusion: Not yet covered by BIA-Obesity**
	Companies should use a nutrient profiling system (government-endorsed and evidence-based, in alignment with nutritional guidelines) to guide their product (re)formulation; in the absence of such system (re)formulation should be aligned with an internationally recognized nutrient profiling system	Exclusion: Already covered by BIA-Obesity
	**Companies should only produce/source fortified food products that are healthy and inherently of high quality in addressing micronutrient deficiencies, and in line with national or regional standards or international guidelines on food fortification (e.g., iron to flour, vitamin A to oil, iodine to salt)[2]**	**Inclusion: Not yet covered by BIA-Obesity**
**Product labeling**	Companies should provide Back-Of-Pack nutritional information in line with Codex Alimentarius standard CAC/GL 2-1985 (e.g., information should be provided as a per 100g or per 100ml basis, or per package if it only contains a single portion)	Exclusion: Already covered by BIA-Obesity
	Companies should adopt an evidence-based Front-Of-Pack nutrient labeling system in line with relevant national, regional and international recommendations and regulations (e.g., Nutri-Score in Europe, warning labels in selected countries)	Exclusion: Already covered by BIA-Obesity

	Nutrition and Health Claims should be used only in line with Codex Alimentarius standards CAC/GL 1-1979 and CAC/GL 23-1997 (this includes the provision that claims should be evidence-based and consistent with national nutrition and health policy)	Exclusion: Already covered by BIA-Obesity
	**Where available, companies should only use an official (government-endorsed) fortification logo to label fortified products[2]**	**Inclusion: Not yet covered by BIA-Obesity**
**Product and brand promotion**	Companies should limit the marketing of less healthy products to any consumer group (including children, adolescents and adults)	Exclusion: Already covered by BIA-Obesity
	Companies should comply with relevant evidence-based public health recommendations on the marketing of foods to children (including the WHO guideline on policies to protect children from the harmful impact of food marketing, among others)	Exclusion: Already covered by BIA-Obesity
	**Companies should comply with 'The International Code of Marketing of Breastmilk Substitutes' and all subsequent related WHA resolutions up to WHA75(21), as well as the Codex Alimentarius 'Code of Hygienic Practice for Powdered Formulae for Infants and Young Children'[3]**	**Inclusion: Not yet covered by BIA-Obesity**
	**Companies should develop, market, and distribute complementary foods only if they meet all relevant national, regional and global standards for composition, safety, quality, nutrient levels, packaging and labeling, and are in line with national dietary guidelines[3]**	**Inclusion: Not yet covered by BIA-Obesity**
	Companies should perform marketing for healthy foods (i.e. foods emphasized by relevant evidence-based guidelines and recommendations, including national food-based dietary guidelines), including marketing activities targeting priority populations (experiencing, or at high risk of malnutrition)[4]	Exclusion: No relevance of this indicator as a key leverage
**Product accessibility**	**Companies should improve the availability and accessibility of healthy foods relative to unhealthy foods, especially for priority populations, e.g., by using existing distribution systems including itinerant sellers and small retailers[5,6]**	**Inclusion: Priority population aspect not yet covered by BIA-Obesity**
	**Companies should improve the affordability of healthy foods relative to unhealthy foods, especially for priority populations[5]**	**Inclusion: Priority population aspect not yet covered by BIA-Obesity**
	**Companies should offer healthy foods in packaging / portion sizes that suit a limited budget to increase the accessibility of these foods for low-income populations**	**Inclusion: Not yet covered by BIA-Obesity**
**Relationships with external organizations**	When requested, companies should support evidence-based forms of engagement between government, academia, civil society and the private sector, e.g., through participation in multi-stakeholder processes with clear and transparent governance mechanisms, including rules of engagement and management of conflict of interest[7]	Exclusion: Already covered by BIA-Obesity
	**Companies should reduce food waste by ensuring that food that cannot be sold is offered at a cheaper price and/or donated to people in need[2]**	**Inclusion: Not yet covered by BIA-Obesity**

**Supply Chain**	**Companies should provide opportunities for small-scale farmers, food processors and traders to access their supply chains, including fair contracts, training and support, and infrastructure investment (considering their overall impact on food security in communities)**	**Inclusion: Not yet covered by BIA-Obesity**
	Companies should work towards a discrimination-free and gender-equal work environment across their supply chain	Exclusion: No particular focus on food environments
	Companies should implement appropriate measures to maintain the nutritional quality of foods along the supply chain (e.g., by avoiding nutrient losses through cold storage facilities and appropriate forms of processing and packaging)	Exclusion: No relevance of this indicator as a key leverage
	**Companies and their suppliers should improve food safety by complying with national regulations and/or the Codex Alimentarius guidelines on General Principles of Food Hygiene**	**Inclusion: Not yet covered by BIA-Obesity**
**Workforce**	**Companies should allow parents to take paid parental leave and provide breastfeeding mothers with appropriate working conditions and facilities at work**	**Inclusion: Not yet covered by BIA-Obesity**
	**Companies should support adequate and healthy diets among employees through appropriate measures (e.g., company cafeterias serving nutritious and healthy food, employee wellness programs, etc.)[8]**	**Inclusion: Not yet covered by BIA-Obesity**
	Companies should increase employment opportunities for workers from priority populations	Exclusion: No particular focus on food environments

[1]Unhealthy ingredient substitutions must not occur, and reformulated unhealthy, highly processed foods / beverages must not be declared 'healthy' or 'healthier'.
[2]This excludes unhealthy, highly processed foods / beverages.
[3]The indicator also covers rules and regulations other than marketing, e.g., regarding the composition, labeling and distribution of products.
[4]This includes complementary foods meeting all relevant national, regional and global standards for composition, safety, quality and nutrient levels, and being in line with national dietary guidelines.
[5]This includes traditional, culturally acceptable foods (7-10), fortified foods (11-14), and commercially available complementary foods (15-17).
[6]This may include measures to reduce waste of fresh, perishable foods.
[7]Some of the included reports from which this recommendation was derived from mention examples which many in the public health community would not consider to be evidence-based, such as company-led consumer education activities; these tend to be older references, e.g., (13, 14, 18).
[8]Some references extend this recommendation to workers that are not direct employees, e.g., suppliers.

Action category	Recommendation for Food and Beverage Manufacturers	Inclusion/Exclusion in Delphi study (with reason)
**Corporate strategy**	The CEO or other senior Executive should be held accountable for leading a strategic response to nutrition within the company's core business, with their remuneration explicitly tied to achieving nutrition-related targets	Exclusion: Already covered by BIA-Obesity

	Companies should identify and address the nutrition-specific needs of priority populations (experiencing, or at high risk of malnutrition) through strategic commercial approaches aligned with national or international guidelines	Exclusion: Already covered by other indicators in Delphi study
**Product formulation**	**Companies should commit to and/or report on increasing the use of locally available and culturally acceptable nutritious foods (e.g., sweet potato, amaranth, millets) in food production**	**Inclusion: Not yet covered by BIA-Obesity**
**Relationships with external organizations**	Companies should publicly disclose names and affiliations of members of their formal panel of experts / names of advisors with expertise on undernutrition and micronutrient deficiencies	Exclusion: No relevance of this indicator as a key leverage
	Companies should publicly disclose a narrative about their stakeholder engagement activities related to malnutrition and priority populations (experiencing, or at high risk of malnutrition)	Exclusion: Already covered by BIA-Obesity
	Companies or their foundations should commit to and/or report on funding non-commercial public health and nutrition programs that serve priority populations (experiencing, or at high risk of malnutrition)	Exclusion: Already covered by BIA-Obesity
**Other**	**For nutrition and active lifestyle programs supported or conducted by the company, all programs should be evidenced-based and aligned with relevant national or international guidelines, and exclude product or brand level branding**	**Inclusion: Not yet covered by BIA-Obesity**
**Action category**	**Recommendation for Retailers**	**Inclusion/Exclusion in Delphi study (with reason)**
**Product labeling**	Retailers should use point-of-purchase displays to influence consumers to make healthier choices	Exclusion: Already covered by BIA-Obesity
**Product accessibility**	Retailers should open retail outlets close to high poverty areas, where they offer healthy foods on credit	Exclusion: Already covered by other indicators in Delphi study
	Retailers should increase their opening hours / offer convenient opening hours	Exclusion: Already covered by other indicators in Delphi study
	Retailers should consider nutritional guidelines in their distribution planning	Exclusion: Already covered by BIA-Obesity
**Supply Chain**	Retailers should conduct demand forecasts to avoid products remaining unsold before the recommended use-by-date	Exclusion: No relevance of this indicator as a key leverage
**Action category**	**Recommendation for Quick-Service Restaurants**	**Inclusion/Exclusion in Delphi study (with reason)**

**Product formulation**	Restaurants should offer boxes for leftovers	Exclusion: No relevance of this indicator as a key leverage
	Restaurants should offer food via buffet organisation	Exclusion: No relevance of this indicator as a key leverage
**Supply chain**	Restaurants should apply waste quantification technologies and managing stocks (e.g., measuring food waste in the kitchen to understand what foods are being wasted and designing a fix, engaging staff to understand the importance of minimizing waste)	Exclusion: No relevance of this indicator as a key leverage

1.3. Information about method consulting

No outside consulting with regard to the chosen methodology took place.

1.4. Indicator codes used during the conduction of the Delphi study

Table s2: Indicator codes used during the conduction of the Delphi study

Indicator ID used during study conduction	Final BIA-DBM ID	Short indicator description
STRAT1-DBM	DBM-STRAT	Corporate strategy
STRAT2-DBM	DBM-SAFET	Food safety
STRAT3-DBM	DBM-FARM	Small-scale farmers
STRAT4.1-DBM	DBM-PARENT	Parental leave
STRAT4.2-DBM	DBM-BREAST	Breastfeeding support
STRAT5-DBM	DBM-WORK	Healthy diets at work
STRAT6-DBM	DBM-CODE	Breastmilk substitutes
STRAT7-DBM	DBM-COMPL	Complementary foods
STRAT8-DBM	DBM-DONAT	Discounts and donations
FORM1-DBM	DBM-INGRED	Increase healthy ingredients
FORM2-DBM	DBM-FORTI	Food fortification
FORM3-DBM	DBM-TRAD	Traditional foods
LABEL1-DBM	DBM-LOGO	Fortification logo
PROMO1-DBM	DBM-PROG	Nutrition programs
ACCESS1-DBM	DBM-SIZE	Packaging and portion size
ACCESS2-DBM	DBM-ACCESS1	Access for priority populations
ACCESS3-DBM	DBM-ACCESS2	Access to fortified foods

2. Code used in the statistical analyses

2.1. R code for individual indicator histograms (first Delphi round)

```
library(HH)
Strat1_DBM <- STRAT1_DBM
HH::likert(...1~., Strat1_DBM, positive.order=FALSE, as.percent = TRUE,
        main="STRAT1-DBM",
        xlab="Percentage",ylab="")
```

The same code structure was applied to create histograms for the remaining indicators.

2.2. R code for final rating histogram (third Delphi round)

```
library(HH)
library(lattice)
library(grid)
library(latticeExtra)
library(multcomp)
library(mvtnorm)
library(survival)
library(TH.data)
library(MASS)
library(gridExtra)
```

```
Histogram_Delphi2 <- Percentages_updatednames

# Modify the names in the data to manually insert line breaks
names(Histogram_Delphi2) <- c("...1", "Strongly in favour\nof exclusion", "In favour of\nexclusion",
"Somewhat in favour\nof exclusion", "Neither in favour of\nexclusion nor inclusion", "Somewhat in
favour\nof inclusion", "In favour of\ninclusion", "Strongly in favour\nof inclusion")
colnames(Histogram_Delphi2)

HH::likert(...1 ~ ., Histogram_Delphi2,
        positive.order = FALSE,
        as.percent = TRUE,
        main = "Histogram",
        xlab = "Percentage",
        ylab = "",
        scales = list(x = list(at = c(-100, -75, -50, -25, 0, 25, 50, 75, 100))),  # Sets labels on both sides
        par.settings = list(
          key = list(text = list(cex = 0.8)) # Adjust cex for text size
        ))
```

3. Additional results

Table s3: Development and (re-)formulation of DBM indicators over the Delphi process; adaptations to the indicators from the previous Delphi round are highlighted in bold

Indicator	Initially proposed indicator based on systematic review	Indicator adaptation based on 1st Delphi round	Indicator adaptation based on 2nd Delphi round	Indicator adaptation based on 3rd Delphi round (final)
**DBM-STRAT** Corporate Strategy *Sectors: M, S, R*	Does the company's commitment to improving population nutrition and health (where it exists) specifically mention undernutrition, micronutrient deficiencies, and/or food insecurity?	Does the company's commitment to improving population nutrition and health (where it exists) specifically mention undernutrition **(wasting, stunting, underweight, micronutrient deficiencies)**, and/or food insecurity?	*NA (No adaptation to the indicator from previous survey)*	Does the company's commitment to improving population nutrition and health (where it exists) specifically mention **addressing** undernutrition (wasting, stunting, underweight, micronutrient deficiencies), **linked to national health and development priorities**?
**DBM-SAFET** Food safety *Sectors: M, S, R*	Does the company (and its suppliers) commit to and/or report on improving food safety by complying with national regulations and/or the Codex Alimentarius guidelines on General Principles of Food Hygiene?	Does the company **[…]** commit to and/or report on **ensuring** food safety by complying with **[…]** the Codex Alimentarius guidelines on General Principles of Food Hygiene?	*NA (No adaptation to the indicator from previous survey)*	*NA (Indicator excluded)*
**DBM-FARM** Small-scale farmers *Sectors: M, S, R*	Does the company commit to and/or report on providing opportunities for small-scale farmers, food processors and traders to access their supply chains, including fair contracts, training and support, and infrastructure investment, considering their overall impact on food security in communities?	Does the company commit to and/or report on providing opportunities for small-scale farmers **[…]** to access their supply chains, including fair contracts, training and support, and infrastructure investment **[…]**?	*NA (No adaptation to the indicator from previous survey)*	*NA (Indicator excluded)*
**DBM-PARENT** Parental leave *Sectors: M, S, R*	Does the company commit to and/or report on allowing parents to take paid parental leave, and to providing breastfeeding mothers with appropriate working conditions and facilities at work?	Does the company commit to and/or report on allowing **all employees (including farm workers and factory labourers)** to take **at least 14 weeks of** paid parental leave, and to providing breastfeeding mothers with appropriate working conditions (**e.g. offering flexible working**	Does the company commit to and/or report on **providing** all employees **[…]** at least 14 weeks of paid **maternity** leave?	Does the company commit to and/or report on providing **primary caregivers** at least 14 weeks of paid **parental** leave?
**DBM-BREAST** Breastfeeding support *Sectors: M, S, R*			Does the company commit to and/or report on providing breastfeeding mothers with appropriate working conditions (e.g., offering flexible working arrangements), and facilities at work	Does the company commit to and/or report on providing breastfeeding mothers with appropriate working **arrangements (e.g., paid nursing breaks)** and facilities at work (e.g., to breastfeed, express and

Indicator	Initially proposed indicator based on systematic review	Indicator adaptation based on 1st Delphi round	Indicator adaptation based on 2nd Delphi round	Indicator adaptation based on 3rd Delphi round (final)
		**arrangements)**, and facilities at work **(e.g. to express and store breastmilk)**?	(e.g., **to breastfeed**, express and store breastmilk)?	store breastmilk), **in line with national, regional or international guidance**?
**DBM-WORK**<br>Healthy diets at work<br>*Sectors: M, S, R*	Does the company commit to and/or report on supporting adequate and healthy diets among employees through appropriate measures (e.g. company cafeterias serving nutritious and healthy food, employee wellness programs, etc)?	Does the company commit to and/or report on **having measures in place to ensure access to** healthy **foods during working hours** (e.g. company cafeterias serving **[…]** healthy foods, **food vouchers which can be used for healthy foods**)?	Does the company commit to and/or report on having measures in place to ensure **that employees can practice a healthy diet at an affordable price** during working hours (e.g., company cafeterias serving **nutritious** foods, food vouchers which can be used for **nutritious** foods)?	*NA (No adaptation to the indicator from previous survey)*
**DBM-CODE**<br>Breastmilk substitutes<br>*Sectors: M, S*	Does the company commit to and/or report on complying with 'The International Code of Marketing of Breastmilk Substitutes' and all subsequent WHA resolutions up to WHA75(21), as well as the Codex Alimentarius 'Code of Hygienic Practice for Powdered Formulae for Infants and Young Children'?	*NA (No adaptation to the indicator from previous survey)*	Does the company commit to and/or report on complying with 'The International Code of Marketing of Breastmilk Substitutes' and all subsequent WHA resolutions **[…]**, as well as the Codex Alimentarius 'Code of Hygienic Practice for Powdered Formulae for Infants and Young Children'?	Does the company commit to and/or report on complying with 'The International Code of Marketing of Breastmilk Substitutes' and all subsequent **related** WHA resolutions, as well as **relevant Codex Alimentarius standards (e.g., CXS 72-1981)**?
**DBM-COMPL**<br>Complementary foods<br>*Sectors: M, S*	Does the company commit to and/or report on developing, marketing, and distributing complementary foods only if they meet all relevant national, regional and global standards for composition, safety, quality, nutrient levels, packaging and labelling, and are in line with national dietary guidelines?	Does the company commit to and/or report on developing, marketing, and distributing **commercial food products for infants and children** only if they **are in line with national evidence-informed recommendations, or (if not available) WHO guidance (e.g. WHO NPPM)**?	Does the company commit to and/or report on **producing**, marketing, and distributing **commercially available complementary foods** only **[…]** in line with national evidence-informed recommendations, or (if not available) **international guidance (e.g. the International Code of Marketing of Breastmilk Substitutes, Codex Alimentarius,** or WHO NPPM**)**?	Does the company commit to and/or report on producing, marketing, **labelling**, and distributing commercially available complementary foods only in line with national evidence-informed recommendations, or (if not available) international guidance (e.g., the International Code of Marketing of Breastmilk Substitutes, Codex Alimentarius **(CXS 156-1987, CAC/GL 8-1991, CXS 73-1981, CXS 74-1981)**, or WHO NPPM)?
**DBM-DONAT**<br>Discounts and donations<br>*Sectors: M, S, R*	Does the company commit to and/or report on reducing food waste by ensuring that food that cannot be sold is offered at a cheaper price and/or donated to people in need?	Does the company commit to and/or report on reducing food waste by ensuring that food that **is considered safe (i.e. within use by date and unspoiled), but that** cannot be sold is offered at a cheaper price and/or donated to people in need?	Does the company commit to and/or report on **applying discounts on nutritious food** that is considered safe (i.e. within **'use-by-date'** and unspoiled) but that cannot be sold **at the regular price**, and/or donating it to people in need?	Does the company commit to and/or report on applying discounts on nutritious food that is considered safe (i.e., within 'use by date' and unspoiled) but that cannot be sold at the regular price, and/or donating it to people in need?

Indicator	Initially proposed indicator based on systematic review	Indicator adaptation based on 1st Delphi round	Indicator adaptation based on 2nd Delphi round	Indicator adaptation based on 3rd Delphi round (final)
**DBM-INGRED**<br>Increase healthy ingredients<br>*Sectors: M, S, R*	What commitments or actions has the company taken with respect to increasing content of fruits, vegetables, legumes, nuts and/or wholegrains in their processed food products? Does the company routinely report on their content across its product portfolio and progress against relevant commitments and best practice standards?	*NA (No adaptation to the indicator from previous survey)*	What commitments or actions has the company taken with respect to increasing content of fruits, vegetables, legumes, nuts and/or wholegrains in their **[…]** food products? Does the company routinely report on **this measure** across its product portfolio and progress against relevant commitments and best practice standards?	*NA (indicator excluded)*
**DBM-FORTI**<br>Food fortification<br>*Sectors: M, S*	Does the company commit to and/or report on only producing/using fortified food products that are healthy and inherently of high quality in addressing undernutrition, and in line with national or regional standards or international guidelines on food fortification (e.g., iron to flour, vitamin A to oil, iodine to salt)?	Does the company commit to and/or report on producing/using**/offering** fortified **or enriched** foods **[…] only** in line with **national, regional, or international standards or recommendations** on food fortification (e.g. iron to flour, vitamin A to oil, iodine to salt)?	**For companies** producing/using**/distributing** fortified or enriched foods, **does the company commit to and/or report on complying with** national, regional, or international standards or recommendations on food fortification **[…]**?	For companies producing/using/distributing fortified or enriched foods, does the company commit to and/or report on complying with **applicable** national, regional, or **(if not available)** international standards or recommendations **(e.g., guidance limiting fortification to nutritious foods, staples and condiments)**?
**DBM-TRAD**<br>Traditional foods<br>*Sectors: M, S, R*	Does the company commit to and/or report on increasing the use of locally available and culturally acceptable nutritious foods (e.g. sweet potato, amaranth, millets) in food production?	Does the company commit to and/or report on increasing the use of **traditional** and culturally acceptable, **safe and healthy** foods (e.g. sweet potato, amaranth, millets) in food production, **in line with national dietary guidelines**?	Does the company commit to and/or report on increasing the use of traditional, culturally acceptable, and **nutritious** foods (e.g. sweet potato, amaranth, millets) in food production, in line with national **or international** dietary guidelines?	Does the company commit to and/or report on increasing the use of traditional foods, that are culturally **acceptable** and nutritious (e.g., sweet potato, amaranth, millets) in **the production of nutritious foods**, in line with national or international dietary guidelines?
**DBM-LOGO**<br>Fortification logo<br>*Sectors: M, S*	Does the company commit to and/or report on only using an official (government-endorsed) fortification logo (where available) to label fortified products?	*NA (No adaptation to the indicator from previous survey)*	*NA (No adaptation to the indicator from previous survey)*	*NA (indicator excluded)*
**DBM-PROG**<br>Nutrition programs<br>*Sectors: M, S, R*	For nutrition and active lifestyle programs supported or conducted by the company, does the company commit to and/or report that all	For nutrition **[…]** programs supported or conducted by the company, does the company commit to and/or report that all programs are evidenced-based	*NA (No adaptation to the indicator from previous survey)*	*NA (indicator excluded)*

Indicator	Initially proposed indicator based on systematic review	Indicator adaptation based on 1st Delphi round	Indicator adaptation based on 2nd Delphi round	Indicator adaptation based on 3rd Delphi round (final)
	programs are evidenced-based and aligned with relevant national or international guidelines, and exclude product or brand level branding?	and aligned with relevant national or international guidelines, and exclude product or brand level branding? **(max. scoring if the company is not supporting / conducting nutrition programs)**		
**DBM-SIZE** Packaging and portion size *Sectors: M, S, R*	Does the company commit to and/or report on offering healthy foods in packaging / portion sizes that suit a limited budget to increase the accessibility of these foods for low-income populations?	*NA (No adaptation to the indicator from previous survey)*	*NA (No adaptation to the indicator from previous survey)*	*NA (indicator excluded)*
**DBM-ACCESS1** Access for priority populations *Sectors: M, S, R*	Does the company commit to and/or report on improving the availability, affordability, and accessibility of healthy foods relative to unhealthy foods for groups experiencing or at high risk of undernutrition and micronutrient deficiencies?	*NA (No adaptation to the indicator from previous survey)*	*NA (No adaptation to the indicator from previous survey)*	*NA (indicator excluded)*
**DBM-ACCESS2** Access to fortified foods *Sectors: M, S*	Does the company commit to and/or report on improving the availability, affordability, and accessibility of fortified essential foods (e.g. dietary oils, salt, sugar), aiming to address micronutrient deficiencies in groups experiencing or at high risk of undernutrition and micronutrient deficiencies?	Does the company commit to and/or report on improving the availability, affordability, and accessibility of fortified **[…]** foods (e.g. dietary oils, salt, sugar**, flour**), **[…] in line with national, regional, or international standards or recommendations on food fortification**?	Does the company commit to and/or report on improving the availability, affordability and accessibility of fortified foods (e.g. dietary oils, salt, **[…]**, flour), in line with national, regional, or international standards or recommendations on food fortification?	*NA (indicator excluded)*

2.3. First Delphi round: assessment of relevance, achievability and measurability

STRAT1-DBM

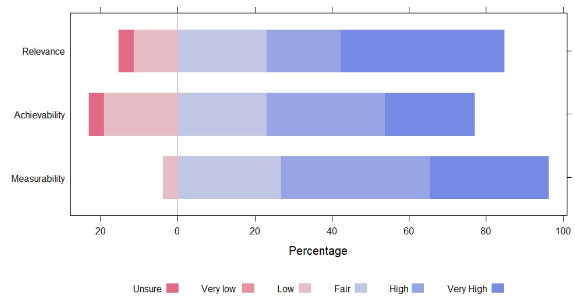

STRAT2-DBM

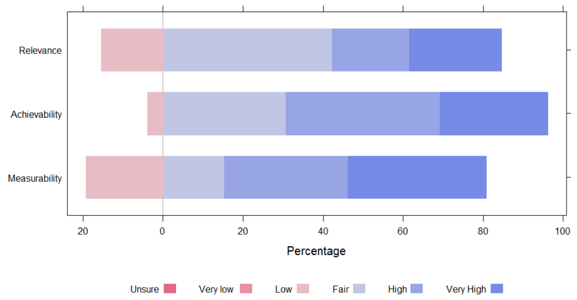

STRAT3-DBM

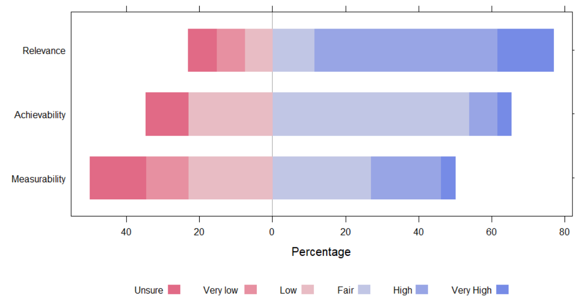

STRAT4-DBM

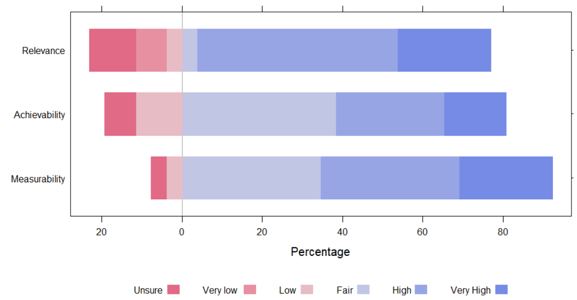

STRAT5-DBM

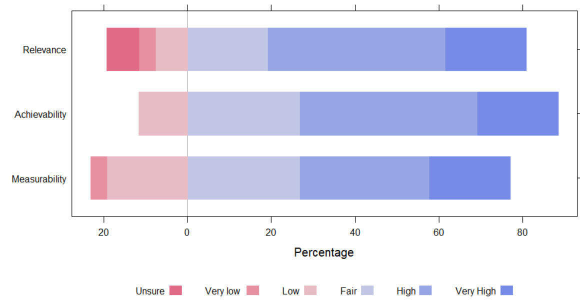

STRAT6-DBM

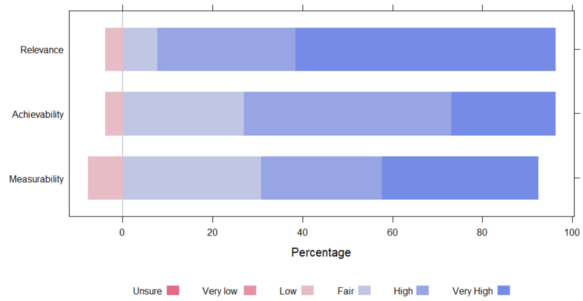

STRAT7-DBM

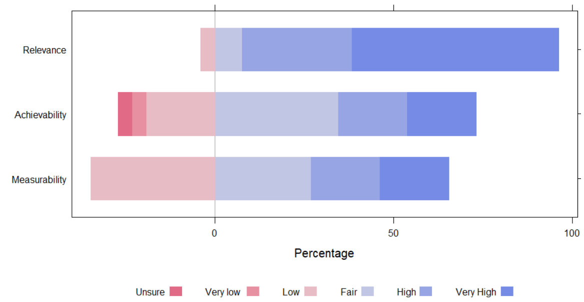

STRAT8-DBM

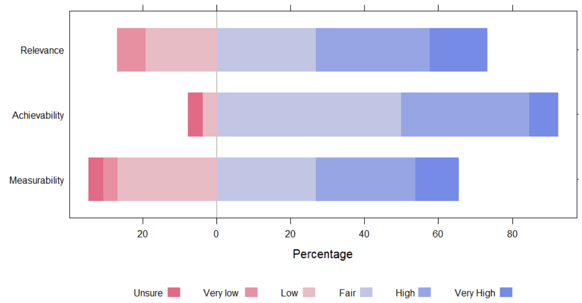

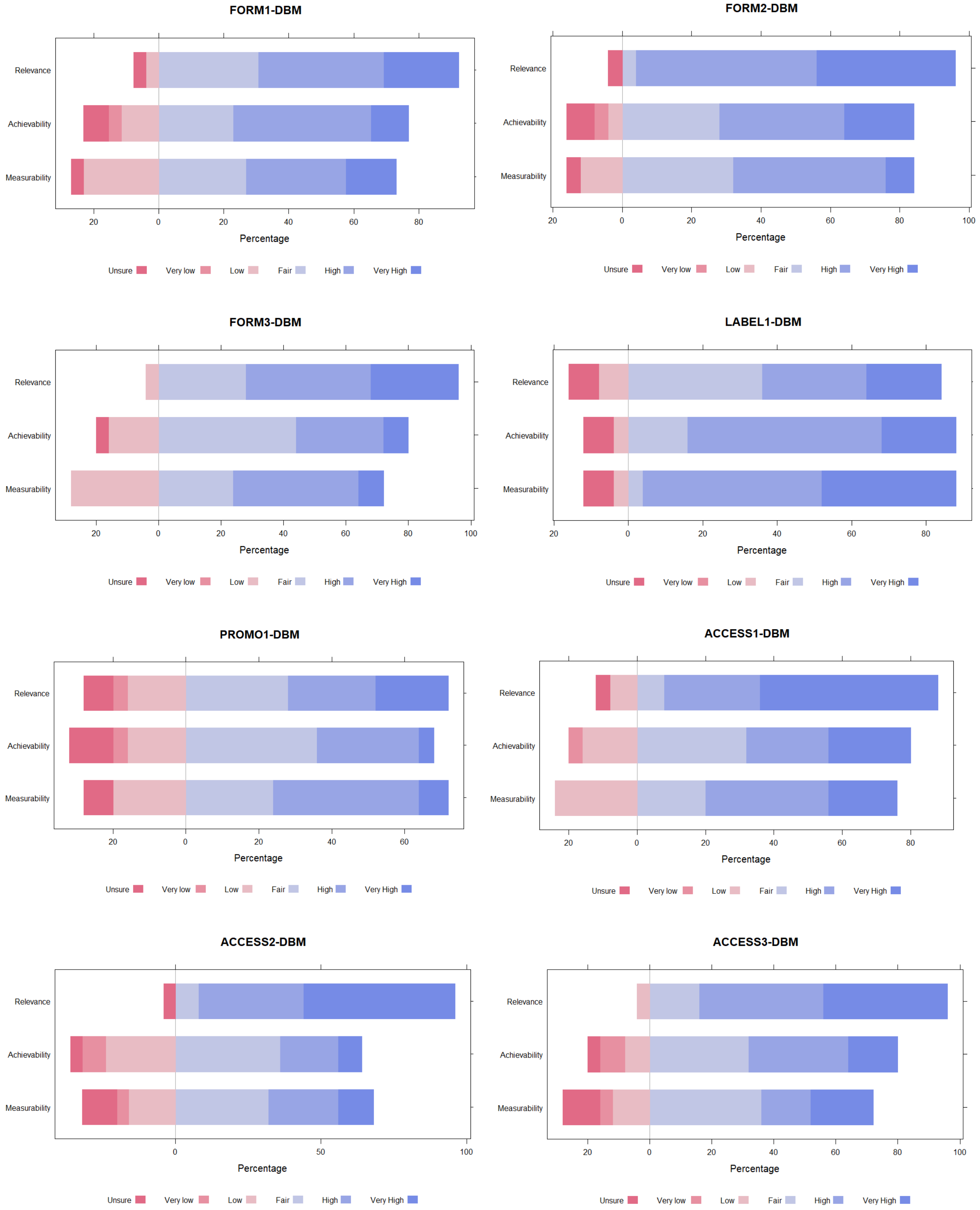

Figure s1: Assessment of indicators' relevance (contribution to addressing undernutrition/DBM), achievability (likelihood of adoption and feasibility of implementation) and measurability (availability of data recorded in a standardized manner) during the first Delphi round

2.4. First Delphi round: qualitative feedback

Table s4: Qualitative feedback provided during the first Delphi round

Feedback from expert panel	How we addressed the feedback
**1. STRAT1-DBM**: Does the company's commitment to improving population nutrition and health (where it exists) specifically mention undernutrition, micronutrient deficiencies, and/or food insecurity?	
1.1 Disaggregate the question for a better understanding (and scoring) of the various aspects (i.e. don't use "and/or"); some people might argue that undernutrition and food insecurity are different concepts	This indicator does indeed cover a number of different aspects. We propose not to split it to keep the total number of indicators manageable (the existing BIA-Obesity tool already includes approx. 80 indicators). We will account for the various aspects through the scoring criteria.
1.2 More relevant if also includes overnutrition and associated problems	Aspects of overnutrition and unhealthy diets are already covered through various indicators of the existing BIA-Obesity tool. The aim of this study is to identify additional indicators particularly targeting undernutrition / the DBM.
1.3 Commitments as published statements easy to measure but relevance and achievability can only be assessed from what is implemented	This is correct and implementation aspects will be covered by step 2 of the BIA-Obesity assessment (step 1 aims to identify and assess only publicly available policies and commitments). However, INFORMAS has responded to this critique by rephrasing some indicators to not only consider existing commitments but also the (public) reporting of actions.
1.4 Unclear if the double burden or triple burden of malnutrition is assessed - clarify/add micronutrient deficiency as part of undernutrition	We follow the WHO definition of a 'double burden of malnutrition', which defines it as undernutrition (wasting and/or stunting and/or underweight and/or micronutrient deficiencies) along with overnutrition (overweight and/or obesity), or diet-related NCDs. The indicator was rephrased incorporating this.
1.5 Instead of an indicator could be a screening question	We interpreted this comment as a suggestion to use this indicator as a criterion whether or not to include a company in the assessment. The overall aim of the BIA-Obesity tool is to assess the most relevant food businesses in a country. Therefore, the respective market share will be used as a selection criterion.

1.6 Companies often mention those keywords on websites, but it is unclear what they are actually doing, i.e. does this only relate to philanthropic actions or also to core business? → mechanism to ensure accountability needed (not just voluntary pledge)	This is correct and implementation aspects will be covered by step 2 of the BIA-Obesity assessment (step 1 aims to identify and assess only publicly available policies and commitments).<br><br>To receive points here, companies' commitments must be aligned with or reference key government or civil society policy documents (including WHO/SDGs/ other recognized reporting standards).<br><br>Specific actions with which the food industry commits/reports to address undernutrition/food insecurity will be assessed as additional indicators within the various domains of the BIA-Obesity framework (product formulation; nutrition labeling; product and brand promotion; product price, affordability, availability and positioning; disclosure of external relationships and lobbying).
1.7 It might be strange to expect quick-service restaurants to address food insecurity	We agree that this indicator may be less relevant for QSRs. However, as shown by some of the more specific indicators, QSRs, too, may be able to take certain actions to address undernutrition and/or food insecurity (e.g. by donating food that is safe but cannot be sold). This is why we suggest applying this indicator to all three sectors.
**2. STRAT2-DBM**: Does the company (and its suppliers) commit to and/or report on improving food safety by complying with national regulations and/or the Codex Alimentarius guidelines on General Principles of Food Hygiene?	
2.1 Grammatically hard to interpret and measure using many 'and/or' phrases - too many nuances	We have reworded the indicator to make it clearer. We also agree that it covers several different aspects, which we will account for through the scoring criteria.
2.2 Food safety is usually well regulated, i.e. businesses should comply with food safety regulations anyway - "easy" way to receive "good" scoring	This is correct; we have therefore removed the reference to national regulations. This indicator would only be used for businesses in countries where there is no binding regulation.
2.3 An important indicator for LMICs but the achievability and measurability of it are challenging, particularly if this includes suppliers as well	We have removed the reference to suppliers to reduce complexity.
2.4 Food industry likes to talk about food safety, often used as distraction from what they are not doing	This is a valid concern. While this indicator would only be one out of approx. 90 indicators that address various issues regarding undernutrition/ overnutrition, we will bring this point up for discussion during the expert workshop.<br><br>*Tbd during consultation workshop*
2.5 Transparent reporting is key here	This will be considered through the scoring.

2.6 Choose a more definite term than "improve" - companies should *ensure* food safety in terms of national regulation and Codex Standards → reword e.g. "does the company have a food hygiene process based on Codex Alimentarius guidelines on General Principles of Food Hygiene or related national legislation?"	The indicator was rephrased incorporating this.
2.7 The relationship with undernutrition/DBM is not very strong or direct	*Tbd during consultation workshop*
**3. STRAT3-DBM:** Does the company commit to and/or report on providing opportunities for small-scale farmers, food processors and traders to access their supply chains, including fair contracts, training and support, and infrastructure investment, considering their overall impact on food security in communities?	
3.1 Too many caveats which make it hard to measure/monitor, e.g. how to measure if a contract is fair? How to monitor infrastructure investment? Should be broken down	At the level of this assessment, the main aim is to check if companies are even aware of this issue and whether they report specific actions. Various aspects will then be captured through the scoring.
3.2 Relevant indicator but unclear to whom this indicator benefits more; the small-scale farmers or the malnourished community?	We have rephrased the indicator to make it more precise (by removing the last part of the sentence).
3.3 Better to differentiate small-scale farmers from food processors and traders (the infrastructure, training, and support may be very different for each one of them)	As small-scale farmers seem to be the most relevant group here, we decided to remove "food processors" and "traders" to make the indicator more precise.
3.4 Indicator relevant mainly for small and medium-sized companies at a local level but not so much for multinationals	We agree that this indicator may be more relevant for small and medium-sized companies; for simplicity, we have decided against developing different indicators for companies of different sizes.
3.5 Commitment to food security and small-scale farmers will not necessarily assist with undernutrition/malnutrition	*Tbd during consultation workshop*
**4. STRAT4-DBM:** Does the company commit to and/or report on allowing parents to take paid parental leave, and to providing breastfeeding mothers with appropriate working conditions and facilities at work?	
4.1 Should be broken down into 2 indicators: 1) parental leave, 2) breastfeeding support in the workplace	To keep the overall number of indicators manageable, we have decided against splitting this indicator. The two aspects will, however, be captured through the scoring criteria.
4.2 Not sure if all companies report on their workplace practices / conditions more generally - failure to report is not indicative of practices	We agree that reporting on this indicator may be less widespread than for others; we would, however, propose to keep it, as the BIA-Obesity framework also aims to encourage companies to start reporting on their practices/conditions if they aren't doing so yet.

4.3 This also depends on national social systems. Comparison between businesses operating in different countries might be tricky	To make this indicator more precise, we have included a reference to the respective Convention of the International Labour Organization (ILO), which recommends at least 14 weeks of maternal leave (ILO Convention No 183). This indicator would only be applicable for countries, where there is no national regulation / social system in place that ensures at least 14 weeks of maternal leave.
4.4 It is important to clarify the length of leave in the indicator; what are the measures in place to ensure that mothers have a minimum period of recovery after delivery especially when employed in the three sectors, which all require heavy physical workload?	We now refer to ILO Convention No 183 (which establishes a minimum of 14 weeks of maternal leave) to address this comment.
4.5 Low relevance/impact. Only affects their own employees, no population-wide impact. Not addressed by original BIA-Obesity. The contribution to malnourishment depends on the socioeconomic distribution of its employees.	*Tbd during consultation workshop*
4.6 This indicator should be applied to two sectors only: food and beverage manufacturers and supermarkets	In our understanding, this indicator might also be relevant to employees of quick-service restaurants, and we would therefore propose to apply it to all three sectors.
4.7 Clarify the term 'appropriate working conditions for breastfeeding mothers'	We have rephrased the indicator accordingly.
4.8 Should be applied to all level of workers (including farm workers and factory laborers)	We have rephrased the indicator accordingly.
**5. STRAT5-DBM:** Does the company commit to and/or report on supporting adequate and healthy diets among employees through appropriate measures (e.g. company cafeterias serving nutritious and healthy food, employee wellness programs, etc.)?	
5.1 Clarify/determine terminology: "adequate and healthy" and "appropriate measures" (giving out pamphlet on nutrition is very different from providing healthy and affordable cafeteria options) - too overarching, difficult to score	We have rephrased the indicator and have removed the wording "appropriate measures". As a common definition for "healthy foods" across all indicators, we propose to use the same approach as the existing BIA-Obesity tool. Hence, we propose to define healthy foods in line with applicable government- or WHO-endorsed dietary guidelines.
5.2 More likely to address overnutrition than undernutrition among employees. Take into account the profiles/rankings of the employees (not all may have the same company benefits)	We have rephrased the indicator accordingly.
5.3 Limited reach and impact, therefore not focus of original BIA-Obesity tool. Is this feasible for the vast majority of companies? (probably just few have their own cafeteria or wellness program). The contribution to malnourishment depends on the socioeconomic distribution of its employees.	*Tbd during consultation workshop*

5.4 In SA - the consumer goods industry employs approx. 20% of the SA workforce, so quite relevant, but maybe not affecting the most marginalized → potentially only include company chains with several structures in the country, i.e. supermarkets and QSRs?	We agree that this indicator may be less relevant for the most marginalized. We will discuss its relevance on a population level during the workshop. We also agree that it may be more relevant for bigger companies but have decided against developing different sets of indicators for companies of different sizes.
5.5 This might be one of the easiest indicators to include and track	Well received, thank you.
**6. STRAT6-DBM:** Does the company commit to and/or report on complying with 'The International Code of Marketing of Breastmilk Substitutes' and all subsequent WHA resolutions up to WHA75(21), as well as the Codex Alimentarius 'Code of Hygienic Practice for Powdered Formulae for Infants and Young Children'?	
6.1 Health education would be needed to ensure companies understand what this means. Unless they produce products that fall under the Code, they may not be aware.	We agree that this indicator is more relevant for companies producing or offering products that fall under the Code. For other companies, it will not be applicable.
6.2 Expensive and difficult to measure at the country level, global policies are easier to check. Mothers could be asked to be the monitors and report where they experienced promotion.	The first step of the BIA-Obesity does not involve an assessment of actual implementation, but rather of the existence of public commitments.
6.3 Compliance with the Code is both relevant and measurable. The achievability depends both on regulations and public pressure (if matters to the consumers).	Well received, thank you. This is the reason why we propose to add this indicator to the tool - to increase public awareness.
6.4 Detailed info might be needed here to interpret broad stoke commitments versus interpreting and scoring the details on how companies read and apply the Code.	We agree, a complete and more detailed assessment is needed to elucidate how companies actually interpret the code. For the BIA-Obesity tool, it is important to keep the assessment doable for small teams of researchers with limited funding. We would therefore propose to include only one indicator on this topic.
**7. STRAT7-DBM:** Does the company commit to and/or report on developing, marketing, and distributing complementary foods only if they meet all relevant national, regional and global standards for composition, safety, quality, nutrient levels, packaging and labelling, and are in line with national dietary guidelines?	
7.1 Too complex to measure. It contains too many elements to make it achievable and/or measurable. Requires several indicators	We have rephrased the indicator to make it clearer.
7.2 Clarify the definition of "complementary foods"	We aligned our wording with the WHO NPPM, which refers to "commercial food products for infants and children".

7.3 The reference to meeting national, regional, and global standards assumes that there is an alignment - but this is not the case for most countries → focus should be on national standards "in line" with global standards.<br><br>National dietary guidelines are however developed for the general population and not specific to the age group 6-23 months, also don't assume a relationship between commercially available baby and toddler foods and FBDG or national dietary guidelines → normally these products are not recommended because are not required/appropriate for healthy weaning	We have rephrased the indicator accordingly. Commercial food products for infants and children should be aligned with national evidence-informed recommendations, or (if not available) guidance developed by the WHO, e.g. the WHO NPPM and the WHO guideline for complementary feeding.
7.4 Complementary foods are often ultra processed and at higher price → even with that labeling may still be harmful to health and contribute to malnutrition	We agree. For this reason, international guidance such as the WHO NPPM (now mentioned in the indicator) recommends limiting marketing of these products.
**8. STRAT8-DBM:** Does the company commit to and/or report on reducing food waste by ensuring that food that cannot be sold is offered at a cheaper price and/or donated to people in need?	
8.1 Food safety regulations/aspects must be considered	We have rephrased the indicator accordingly, adding the qualifier that the food should be safe.
8.2 More important for environmental sustainability than for undernutrition - no direct link	*Tbd during consultation workshop*
8.3 Quality of food needs to be considered; report the nutritional information of what is donated	Issues of food safety have been incorporated into the indicator. We suggest considering the nutritional quality of the food through the scoring.<br><br>*Tbd during consultation workshop*
8.4 Capture the difference between overall/global commitments versus local pilots/ad hoc initiatives	We suggest addressing this as part of the scoring.
8.5 Also consider food waste reduction strategies before this point, e.g. through supply chain factors (storage, transport, etc.) - might also require softening of existing policies/systems (e.g. favoring "best before" instead of "use by")	We agree that food waste reduction strategies along the supply chain are important for food waste reduction overall; however, they may be only indirectly linked to undernutrition and food security. We would therefore propose to limit the indicator to food waste at the retail / food service level.
**9. FORM1-DBM:** What commitments or actions has the company taken with respect to increasing content of fruits, vegetables, legumes, nuts and/or wholegrains in their processed food products? Does the company routinely report on their content across its product portfolio and progress against relevant commitments and best practice standards?	
9.1 This indicator could be challenging for companies due to competition law; in EU it is not allowed to communicate reformulation issues until relatively large changes have been implemented (e.g. 30% reduction of salt)	To our knowledge, the corresponding EU regulation is only applicable to promotional / labeling aspects. Companies are still allowed to publish reformulation goals below this threshold as part of their reporting.

9.2 “Positive reformulation” / “nutri-washing” (adding big amounts of wholegrain, micronutrients, and other healthy ingredients to UPFs); can be a distraction from the industry’s lack of progress in reducing risky nutrients → therefore not included in original BIA-Obesity<br><br>Ensure content is elevated with levels that contribute to consumer's nutrition versus additions that are done primarily for flavoring or marketing claims	We agree that commitments of this kind could be misused to distract from lack of progress in reducing problematic nutrients. It could, however, be argued that in contexts with a substantial burden of undernutrition and micronutrient deficiencies, this kind of "positive reformulation" is more important than in contexts without such a burden.<br><br>*Tbd during consultation workshop*
9.3 The relevance for undernutrition and DBM would depend also on the level of processing and inclusion of additives → the measurability should be ensured through food labeling	We agree that the level of processing is a crucial aspect. Insofar as national dietary guidelines recommend to limit the consumption of ultra-processed foods, this aspect will be covered within the ACCESS domain (access to healthy/healthier vs. less healthy foods).
9.4 Needs to be complemented with a pricing strategy to not increase costs of such foods and should also be accompanied by a commitment to reduce harmful ingredients; desirability should be considered as well.	We agree that affordability, pricing, content of problematic ingredients and desirability are crucial aspects. These aspects are covered by several existing indicators of the BIA-Obesity tool (M-ACCESS2, S-ACCESS1, R-ACCESS1, M-FORM2-5, S-FORM2-5, R-FORM2-5).
9.5 The FVNL is a difficult one to actually measure/capture in many markets where it isn't listed on the nutrition facts label, but still relevant to try to capture. It may also be necessary to break these down further to two sub-indicators for FVNL and whole grains. Also, may be difficult to distinguish commitments to increase fibre and increase whole grains?	We agree that this indicator may be difficult to assess on the level of actual implementation (in the first step of the BIA-Obesity assessment, only public commitments are assessed).<br><br>To keep the overall number of indicators manageable, we have decided against splitting this indicator. The two aspects will, however, be captured through the scoring criteria.
**10. FORM2-DBM:** Does the company commit to and/or report on only producing/using fortified food products that are healthy and inherently of high quality in addressing undernutrition, and in line with national or regional standards or international guidelines on food fortification (e.g. iron to flour, vitamin A to oil, iodine to salt)?	
10.1 The definition of “healthy” and “high quality” should be clarified to avoid misinterpretation. Also differentiate the term “fortification” and “enrichment”.	We have rephrased the indicator, excluding the terms “healthy” and “high quality” from the indicator formulation, as many staple foods (white flour, oil, sugar, salt) are not considered healthy, but may still be candidates for fortification according to relevant guidelines and standards.<br><br>We have added a reference to enrichment.
10.2 Unlikely that a company commits to solely producing this type of product	There might have been a misunderstanding based on the wording of the indicator. We do not propose that all products must be fortified but that fortification should only be done in line with widely recognized standards. We have rephrased the indicator to address this.

10.3 Possible within manufacturers, more challenging in supermarkets and QSRs	We agree that this indicator will be more relevant for food manufacturers than for retailers and QSRs. However, many retailers offer own-brand products, and QSR may use fortified products as ingredients (e.g. iodized salt).
10.4 Achievability might be hard to accomplish. Depends a lot on national policies and standards for fortification	We agree that achieving full implementation may be challenging for this indicator. Of note, it would only be applicable for countries, where there is no binding regulation regarding the fortification of particular foods in place.
10.5 Independent testing of content of products e.g. iron to flour, vitamin A to oil, iodine to salt as opposed to committing to and/or report on would be more rigorous	We agree that independent testing would be more rigorous than an assessment of public commitments. Implementation aspects are assessed as part of the second step of applying the BIA-Obesity tool.
10.6 Indicator highly relevant in countries facing micronutrient deficiencies. Can be considered as a context-specific indicator	We agree that this indicator (as well as several others) is context-specific.
10.7 Suggestion to restrict this indicator to staple foods and condiments to be of most relevance to undernutrition and DBM	*Tbd during consultation workshop*
**11. FORM3-DBM:** Does the company commit to and/or report on increasing the use of locally available and culturally acceptable nutritious foods (e.g. sweet potato, amaranth, millets) in food production?	
11.1 More relevant for overall sustainability than nutrition	We replaced ‘local’ with ‘traditional’ to make it more precise.<br><br>*Tbd during consultation workshop*
11.2 Easier to measure but achievability would depend on local agriculture. Also, good regulation of agrochemicals and aflatoxin testing needed to ensure a safe product.	We incorporated this feedback by adding “safe” to the wording of the indicator.
11.3 For measurability and cultural acceptability consider alignment with national dietary guidelines and dietary surveys.	We agree and have rephrased the indicator accordingly.
11.4 Maybe not applicable to all food categories. May need to limit to products in particular categories.	We agree that this indicator might not be applicable to all food categories, which may be considered as part of the assessment.
11.5 Also use “healthy” instead of “nutritious” here and find a common definition for “healthy” across all indicators.	We agree and have rephrased the indicator accordingly. As a common definition for "healthy foods" across all indicators, we propose to use the same approach as the existing BIA-Obesity tool. Hence, we propose to define healthy foods in line with applicable government- or WHO-endorsed dietary guidelines.
11.6 Only limited to crops, or also including animal products?	This would depend on the respective national dietary guideline, but could, in principle, also include animal products.

11.7 How would 'local' be defined for a national or international supermarket chain?	We replaced ‘local’ with ‘traditional’ to make it more precise.
**12. LABEL1-DBM:** Does the company commit to and/or report on only using an official (government-endorsed) fortification logo (where available) to label fortified products?	
12.1 This should apply to largely consumed staple food and condiments.	*Tbd during consultation workshop*
12.2 The indicator seems to overlap with the indicator on food fortification<br><br>Are both necessary?	Both refer to food fortification - one focuses on the formulation, the other on the labeling of these products.
12.3 In India some producers do fortify but don't use the logo because they are afraid consumers don't want/trust fortified foods.	The aim of this indicator is to ensure that **if** a fortification logo is used, it should be an official (government-endorsed) logo and not one that was created by the company itself (if an official logo exists).
12.4 Unclear how it would be applied to supermarkets other than their role as a manufacturer?	For supermarkets, this would indeed be only applicable for the supermarkets’ own brand products.
**13. PROMO1-DBM:** For nutrition and active lifestyle programs supported or conducted by the company, does the company commit to and/or report that all programs are evidenced-based and aligned with relevant national or international guidelines, and exclude product or brand level branding?	
13.1 Only include nutrition programs - active lifestyle programs can be a distraction from the responsibility of nutrition practices; this should be supported by other measures directly linked to their business activities.	We agree and have rephrased the indicator accordingly.
13.2 Too complex as a single measure, need to be broken down into i) evidence-based, ii) alignment, iii) no branding	We agree that these aspects should be considered separately, which will be done through the scoring.
13.3 Here only transparency should be assessed → not the role of companies to develop and implement such programs - ongoing discussion;<br><br>High risk of conflict of interest if a company conducts nutrition and active lifestyle programs or if it funds those through third parties to influence either the public, the government or research	*Tbd during consultation workshop*
13.4 The company should also commit to not using external marketing campaigns to gain public endorsement / favorable public optics, deflecting from their perhaps otherwise harmful products	The BIA-Obesity tool includes a number of indicators on marketing of unhealthy products towards children. Demanding that companies commit to not using any marketing campaigns at all may not be realistic.
**14. ACCESS1-DBM:** Does the company commit to and/or report on offering healthy foods in packaging / portion sizes that suit a limited budget to increase the accessibility of these foods for low-income populations?	
14.1 Nice idea, great addition. This could be part of their basket of offerings, making it more palatable.	Well received, thank you.

14.2 This indicator is risky → small portions of healthy food can be more expensive per weight unit	We agree that this aspect can be problematic. However, in very resource-constrained settings, smaller portion sizes may help to improve accessibility.<br><br>*Tbd during consultation workshop*
14.3 "Healthy foods" should be defined, e.g. add "fresh and/or minimally processed" to "healthy food" or include examples (e.g. fruits, vegetables, legumes, animal source food, nuts, whole grains, etc.)	We agree that the term "healthy" needs to be defined. As a common definition for "healthy food" across all indicators, we propose to use the same approach as the existing BIA-Obesity tool. Hence, we propose to define healthy foods in line with applicable government- or WHO-endorsed dietary guidelines.
14.4 More achievable if all / many companies agree to a range of products that are made affordable and not just few companies to reduce initial risk	We agree that there may be such spill-over effects, which could ideally be set off by the BIA-Obesity assessment.
**15. ACCESS2-DBM:** Does the company commit to and/or report on improving the availability, affordability, and accessibility of healthy foods relative to unhealthy foods for groups experiencing or at high risk of undernutrition and micronutrient deficiencies?	
15.1 Very clear definitions and/or examples of "healthy" and "unhealthy" are needed → e.g. addition "fresh and/or minimally processed healthy foods" (e.g. fruits, vegetables, legumes, animal source food, nuts, whole grains, etc.)	We agree that the term "healthy" needs to be defined. As a common definition for "healthy food" across all indicators, we propose to use the same approach as the existing BIA-Obesity tool. Hence, we propose to define healthy foods in line with applicable government- or WHO-endorsed dietary guidelines.
15.2 Why only for undernutrition and micronutrient deficiencies?<br><br>This indicator could potentially be relevant to the entire population.	The original BIA-Obesity tool already includes indicators (ACCESS1, ACCESS2) with regards to availability and affordability targeting the general population and not with a particular focus on vulnerable groups.<br><br>*Tbd during consultation workshop*
15.3 How to identify the target groups?<br><br>Consider privacy issues and stigmatization. Unlikely that commercial companies would implement measures that demand a lot of logistics.	*Tbd during consultation workshop*
15.4 Reference to availability and affordability enough → both leads to better accessibility	Availability and affordability are indeed related to accessibility; however, in our understanding, accessibility also includes additional aspects (e.g. in-store placement). We would therefore propose to keep it.
15.5 Desirability should be considered as well.	*Tbd during consultation workshop*
**16. ACCESS3-DBM:** Does the company commit to and/or report on improving the availability, affordability, and accessibility of fortified essential foods (e.g. dietary oils, salt, sugar), aiming to address micronutrient deficiencies in groups experiencing or at high risk of undernutrition and micronutrient deficiencies?	

16.1 How to identify the target groups?<br><br>Consider privacy issues and stigmatization. Unlikely that commercial companies would implement measures that demand a lot of logistics.	*Tbd during consultation workshop*
16.2 This measure (fortification of oils and sugar) might exacerbate overweight and obesity in the same population. Might be contradictory to previous indicator, that states that only “healthy” foods should be fortified.<br><br>Increasing access to dietary oils, salt and sugar, even if fortified, does not seem like a sensible solution in terms of the DBM.	*Tbd during consultation workshop*
16.3 Combining availability, affordability, and accessibility in one question will make the analysis and interpretation for companies difficult	We agree that these are different aspects, which we will address through the scoring criteria. We would propose to refrain from splitting the indicator to keep the overall number of indicators manageable.
**17. Further comments and suggestions**	
17.1 Other questions might merit discussion: 1) how to assess company performance when businesses operate under different jurisdictions? 2) Is there a risk we assign credits when companies simply do not break rules?	These are valid considerations. Indicators will only be applicable for businesses in countries where there is no binding regulation regarding that issue.
17.2 Add an indicator on whether companies respect the human right to adequate food, and follow-up question on whether they perform a due diligence process to "know and show" whether their operations influence peoples' right to food negatively	Thank you for this suggestion. We believe with adding STRAT1-DBM, ACCESS1-DBM and ACCESS2-DBM to the current BIA-Obesity tool (and together with STRAT1 and STRAT2 of the original tool), this aspect should be covered. To keep the overall number of indicators manageable, we have decided against adding additional indicators on that issue.
17.3 Be careful with the use of “healthy” an “unhealthy” (very subjective) → define a classification system (nutritional criteria) to be used by food companies	We agree that the term "healthy" needs to be defined. As a common definition for "healthy food" across all indicators, we propose to use the same approach as the existing BIA-Obesity tool. Hence, we propose to define healthy foods in line with applicable government- or WHO-endorsed dietary guidelines.
17.4 Some indicators may apply only to large-scale companies. Consider the sectors that you are including - for example, low-income populations may not use supermarkets but rather smaller shops to procure their food, many of which don't offer fresh produce.	This is a very valid point. However, the companies that will be assessed with the BIA-DBM tool are selected based on their market share (as it is expected that they have a big effect on population nutrition when changing their practices). Hence, small companies / shops will not be part of the assessment but will be targeted by WP5 of the FoodSAMSA project focusing on the informal food sector.

17.5 Unpacking more of the 'commercial determinants of health' aspects and tactics used may be useful - e.g. lobbying, funding research, reporting of conflicts of interest, and power dynamics	Those aspects are partly considered under domain 6 of the BIA-Obesity tool "Disclosure of external relationships" and will be further assessed using another INFORMAS tool "Monitoring food industry's corporate political activity"
17.6 Measurability needs to be a key consideration. Some aspects require being vague, as laws and contexts differ by country and industry but ensure that technical wording cannot be manipulated by industry (intentionally or unintentionally). Some sort of accountability system would be worth considering, e.g. social audits.	Thank you for this valuable comment. We hope that via this Delphi process we will agree on relevant indicators addressing undernutrition / the DBM whose scoring cannot be manipulated by the food industry. Implementation will be assessed in a second step of the BIA-Obesity assessment.
17.7 Adopt an informative logo on the nutritional quality of foods to guide consumer choice.	Thank you for this suggestion. The adoption of a voluntary government-endorsed front-of-pack scheme already represents one indicator in the original BIA-Obesity tool (M-LABEL6, S-LABEL6).
17.8 Many Big Food companies contribute to food insecurity through the clearing of forests, exploitation of land and resources contributing to food insecurity in the countries of production → room for an indicator on sustainable and ethical production methods?	Thank you for these valid considerations. This is very true, however, we would like to keep the overall number of indicators manageable. As this aspect would mainly address sustainability, and only indirectly affects population nutrition, we have decided against adding an additional indicator on that issue.
17.9 Commitment by food companies to promote only healthy foods with educational messages and this applies most to supermarkets probably - do they support healthy food subside/ voucher programmes Eg SNAP in the USA, Healthy Start in the UK.	Thank you for this suggestion. Such programs would already be covered by some indicators of the original BIA-Obesity tool: S-PROMO7 - S-PROMO9.

2.5. Second Delphi round: qualitative feedback

Table s5: Qualitative feedback provided during the second Delphi round (consultation workshop)

**Feedback from expert panel**	**How we addressed the feedback**
**1. STRAT1-DBM**: Does the company's commitment to improving population nutrition and health (where it exists) specifically mention undernutrition (wasting, stunting, underweight, micronutrient deficiencies), and/or food insecurity?	
This indicator was not discussed during the workshops as we received only few comments on it during the first online survey round.	
**2. STRAT2-DBM**: Does the company commit to and/or report on ensuring food safety by complying with the Codex Alimentarius guidelines on General Principles of Food Hygiene?	
2.1 BIA-Obesity focuses on the biggest food companies / multinationals; will supposably ensure the safety of their products in all countries of operation	*Based on these comments we would propose not including this indicator.*
2.2 In LMICs, rather implementation / enforcement issues, instead of not having food safety regulations in place	

2.3 Indicator probably not applicable to many countries due to existing national food safety standards (based on Codex)	
**3. STRAT3-DBM:** Does the company commit to and/or report on providing opportunities for small-scale farmers to access their supply chains, including fair contracts, training and support, and infrastructure investment?	
3.1 Relevance depends on the country context / amount of people working as small-scale farmers	*We did not find a good way to address the above comments by rephrasing the indicator but acknowledge these issues may be reasons to not include the indicator. We would like to leave this decision to the expert panel.*
3.2 Could be misused as a CPA (corporate political activity) strategy → this could be addressed by e.g. adding rigor to the scoring criteria (at least 40% of supply chain being small-scale farmers)	
3.3 Could change wording to "fresh food" farmers to ensure nutritional quality; small-scale farmers could also produce unhealthy products, e.g. palm oil	
3.4 Political question underlying this; many people supporting small-scale farmer movements would not agree with encouraging companies to incorporate them in their supply chains	
**4. STRAT4-DBM:** Does the company commit to and/or report on allowing all employees (including farm workers and factory laborers) to take at least 14 weeks of paid parental leave, and to providing breastfeeding mothers with appropriate working conditions (e.g. offering flexible working arrangements), and facilities at work (e.g. to express and store breastmilk)?	
4.1 Limit this indicator to paid leave of mothers (fathers not playing a role when it comes to breastfeeding and they do not need to recover from birth); potentially add parental leave as a separate indicator	*We split the previous indicator (STRAT4-DBM) into two indicators (1. maternal leave, 2. breastfeeding) and rephrased the two parts according to the feedback provided.*
4.2 Massive outsourcing of labor to labor brokers in the food industry (companies not having authority about informal labor); more realistic to apply to a particular group (e.g. factory workers), or cover this as part of scoring criteria (if all or only part of employees will be covered)	
4.3 Covers aspect of company culture change / innovation / transformation pathways - shows that company is committed to a healthy workforce	
4.4 Many countries already have some sort of maternity leave in place and usually governments pay for maternity leave. Some companies offer top-ups to match (a share of) the previous salary; length of leave and top-ups could be considered through scoring criteria	
4.5 Quite a small number of people that will be reached by this indicator	
**5. STRAT5-DBM:** Does the company commit to and/or report on having measures in place to ensure access to healthy foods during working hours (e.g. company cafeterias serving healthy foods, food vouchers which can be used for healthy foods)?	
5.1 Include aspect of equitable pricing (healthy vs. unhealthy) into indicator formulation	*We have rephrased the indicator based on these comments, but*

5.2 Unlikely that quick-service restaurants would offer staff healthier food than they are serving; few companies have canteens, mostly unhealthy food environments around their premises	*acknowledge the above issues may be reasons to not include the indicator. We would like to leave this decision to the expert panel.*
5.3 Relevance depends on how many people in a country are employed by the food industry	
5.4 Could be a way of educating through example, creating a culture change (from the inside)	
5.5 Way for companies to artificially boost their score; difficult to measure	
**6. STRAT6-DBM:** Does the company commit to and/or report on complying with 'The International Code of Marketing of Breastmilk Substitutes' and all subsequent WHA resolutions up to WHA75(21), as well as the Codex Alimentarius 'Code of Hygienic Practice for Powdered Formulae for Infants and Young Children'?	
This indicator was not discussed during the workshops as we received only few comments on it during the first survey round.	
**7. STRAT7-DBM:** Does the company commit to and/or report on developing, marketing, and distributing commercial food products for infants and children only if they are in line with national evidence-informed recommendations, or (if not available) WHO guidance (e.g. WHO NPPM)?	
7.1 'The Code' already covers the marketing of complementary foods (WHA 69.8) but not their production or distribution	*We have rephrased the indicator based on these comments.*
7.2 Regulation of complementary foods through Codex Alimentarius, not the WHO NPPM (the latter more stringent)	
7.3 Rephrase to "commercially (available) complementary foods" - leave out "for infants and young children" to avoid assumption that these foods are necessary	
7.4 National dietary guidelines usually start from 2 years onwards and don't address age group 6-23 months	
**8. STRAT8-DBM:** Does the company commit to and/or report on reducing food waste by ensuring that food that is considered safe (i.e. within use by date and unspoiled), but that cannot be sold is offered at a cheaper price and/or donated to people in need?	
8.1 This food should not only be safe but also fresh and healthy (no packaged, unhealthy foods); taking into account a double/triple burden approach	*We have rephrased the indicator based on these comments.*
8.2 Bigger political food system issues - if companies offered affordable healthy food, we might not even need this indicator	
8.3 Selling ugly fruits / vegetables at a cheaper price would fit into this category (but could also be separate indicator on its own)	
8.4 Indicator should rather focus on food insecurity instead of reducing food waste	
**9. FORM1-DBM:** What commitments or actions has the company taken with respect to increasing content of fruits, vegetables, legumes, nuts and/or wholegrains in their processed food products? Does the company	

routinely report on their content across its product portfolio and progress against relevant commitments and best practice standards?	
9.1 Consider unintended consequences: increase of sugar / fat content - currently no published limits of these ingredients for packaged foods that could be referred to	*We did not find a good way to address the above comments by rephrasing the indicator, but acknowledge that these issues may be reasons to not include the indicator. We would like to leave this decision to the expert panel.*
9.2 Increasing the content of these food groups in processed food is not solving the issue - still processed foods; a lot of ingredients' benefits get lost during processing (bioavailability of micronutrients, fiber,...)	
9.3 This indicator could be misused for marketing / CSR purposes (particularly the word "increasing"), without actually improving the nutritional quality of the product	
9.4 Transitional pathway: are companies doing something or not?	
9.5 Relevance might be higher for undernutrition/DBM context	
9.6 Almost impossible to retrieve this information from a label / nutrient profile model (for monitoring purposes)	
**10. FORM2-DBM:** Does the company commit to and/or report on producing/using/offering fortified or enriched foods only in line with national, regional, or international standards or recommendations on food fortification (e.g. iron to flour, vitamin A to oil, iodine to salt)?	
10.1 "selling"/"distributing"/"retailing" better than "offering"	*We addressed the general concerns regarding fortification by rephrasing the indicator to make it explicit that we do not recommend fortification in principle but if a company fortifies products this should be done in line with existing standards / regulations.*
10.2 Restriction to staples and condiments highly consumed by a specific population - avoid that people think if they eat fortified (processed) foods, they won't have to eat other micronutrient rich food; might lead to exceeding the safety level of micronutrient intake if also processed foods are being fortified; what if condiments are unhealthy (e.g. Knorr cube being high in salt/sodium)?	
10.3 Might contradict the market access of more natural foods and the home harvesting and grinding of nutritious foods	
**11. FORM3-DBM:** Does the company commit to and/or report on increasing the use of traditional and culturally acceptable, safe and healthy foods (e.g. sweet potato, amaranth, millets) in food production, in line with national dietary guidelines?	
11.1 Suggestion to use "native" (= local/from the area) instead of "traditional" → "traditional" relates more with diets and "native" relates more with crop → however FAO publication on "traditional foods"	*We have rephrased the indicator based on these comments.*
11.2 Rather use "nutritious" foods → "healthy" only used when referring to food groups or diets	
11.3 "safe" not necessary here - all foods should be safe; on the other hand: aflatoxins etc. huge driver of underweight and stunting (but Codex also includes levels of aflatoxins etc.)	
11.4 Relevance depends on processed nature of these foods (unprocessed vs. processed millets)	

<table>
<tr><td>11.5 Applicability issue: In African countries multinationals dominating the market (do not necessarily have production facilities in the countries) and traditional foods often only small production (might not be able to compete with requested amounts)</td><td rowspan="2"></td></tr>
<tr><td>11.6 Retailers have growing power to decide what goes in on the shelves as the own brand increases (future proof of the tool going forward)</td></tr>
<tr><td colspan="2">12. LABEL1-DBM: Does the company commit to and/or report on only using an official (government-endorsed) fortification logo (where available) to label fortified products?</td></tr>
<tr><td colspan="2">This indicator was not discussed during the workshops as we received only few comments on it during the first survey round.</td></tr>
<tr><td colspan="2">13. PROMO1-DBM: For nutrition programs supported or conducted by the company, does the company commit to and/or report that all programs are evidenced-based and aligned with relevant national or international guidelines, and exclude product or brand level branding? (max. scoring if the company is not supporting / conducting nutrition programs)</td></tr>
<tr><td>13.1 “Nutritional programs” is too broad and has to be defined more clearly</td><td rowspan="4">Given these considerations and the fact that there is already an existing RELAT indicator on the disclosure of nutrition education / healthy diet oriented programs, we propose not including this indicator.</td></tr>
<tr><td>13.2 Used as marketing activity by companies - therefore suggestion to only focus on disclosure and transparency of these actions</td></tr>
<tr><td>13.3 Potentially an indicator to give out minus points → zero points if companies don’t do it at all and minus points if they do it in a non evidence-based way and/or with branding</td></tr>
<tr><td>13.4 Creates various, serious conflicts of interest beyond the marketing issue</td></tr>
<tr><td colspan="2">14. ACCESS1-DBM: Does the company commit to and/or report on offering healthy foods in packaging / portion sizes that suit a limited budget to increase the accessibility of these foods for low-income populations?</td></tr>
<tr><td>14.1 Incorporate equitable pricing aspect, e.g. “price per weight unit should remain the same” OR “packaging / portion sizes at similar prices that suit a limited budget…”; instead of including in the wording, could be part of scoring, as price aspects are also covered by other indicators already and because the main aim of the indicator is to offer people different buying strategies at various times per month/year (even at a slightly higher price)</td><td rowspan="3">We would keep the focus of this indicator on providing various packaging / portion size options and would consider the price aspect as part of the scoring criteria.</td></tr>
<tr><td>14.2 Might not be realistic for supermarkets as the producers produce different packing / portion sizes and control the price</td></tr>
<tr><td>14.3 Does this actually improve access? (in South Africa, low-income populations usually get access to these foods by buying in bulk at the end of the month)</td></tr>
<tr><td colspan="2">15. ACCESS2-DBM: Does the company commit to and/or report on improving the availability, affordability, and accessibility of healthy foods relative to unhealthy foods for groups experiencing or at high risk of undernutrition and micronutrient deficiencies?</td></tr>
</table>

<table>
<tr><td>15.1 Important to have an indicator particularly focusing on that target group, maybe use “nutritionally vulnerable group” instead</td><td rowspan="4">Given these considerations and the fact that there is already an existing ACCESS indicator that includes geographic distribution considerations, we propose not including this indicator as the added value may be limited.</td></tr>
<tr><td>15.2 Use healthy food groups vs. unhealthy food groups instead of “foods”</td></tr>
<tr><td>15.3 Incorporate geographical distribution across regions and areas (that normally have less access to these foods)</td></tr>
<tr><td>15.4 Multinationals have different standards when it comes to nutritional quality depending on HICs or LMICs - is this also covered by this indicator or should we create another indicator for this?</td></tr>
<tr><td colspan="2">16. ACCESS3-DBM: Does the company commit to and/or report on improving the availability, affordability, and accessibility of fortified foods (e.g. dietary oils, salt, sugar, flour), in line with national, regional, or international standards or recommendations on food fortification?</td></tr>
<tr><td>16.1 Food fortification as medium-term strategy, long-term strategy should be dietary diversity</td><td rowspan="6">Based on these comments and given that fortification aspects are already covered by indicator FORM2-DBM, we would propose not including the indicator.</td></tr>
<tr><td>16.2 In the community (including WHO) and in the literature, it is often argued that in the medium-term the fortification of essential foods is the most feasible strategy to address micronutrient deficiencies</td></tr>
<tr><td>16.3 Might perpetuate the nutritional situation and the overuse of starches, oils, salt, etc.</td></tr>
<tr><td>16.4 Look at level of processing - with these ingredients, mostly end up being UPFs</td></tr>
<tr><td>16.5 Salt would always have to be fortified with folate, even in the long term, as the amount might not be reached even following a diverse diet</td></tr>
<tr><td>16.6 Supplementation of micronutrients should be a health system issue not a food system issue</td></tr>
<tr><td colspan="2">17. Further comments and suggestions</td></tr>
<tr><td colspan="2">17.1 To avoid companies trying to score better in assessment by achieving more points in DBM indicators, consider reporting the two parts separately → all DBM indicators in one extra domain</td></tr>
<tr><td colspan="2">17.2 In Food-EPI adaptation, additional indicators were really on DBM, not only on undernutrition - this would be a separate tool. New DBM indicators should try to address both, undernutrition and overnutrition at the same time</td></tr>
<tr><td colspan="2">17.3 Introduce a new domain looking at employees and the working environment instead of having those indicators under the corporate nutrition strategy domain → consider new sub-domain?</td></tr>
<tr><td colspan="2">17.4 Not necessary to think about every nuance, indicators can/should still be adapted to the country context prior to implementation</td></tr>
<tr><td colspan="2">17.5 In BIA-Obesity, it is all about processed and packaged foods → no indicators about processing included; tool is not about revolutionizing the food system, it’s about incremental change</td></tr>
</table>

2.6. Third Delphi round: final rating regarding the inclusion of indicators

Table s6: Final rating regarding the inclusion of proposed indicators during the third Delphi round; consensus was defined as a group agreement of 75% or higher (in bold) – this includes Likert scale ratings from 5 – 'somewhat in favour of inclusion' to 7 – 'strongly in favour of inclusion'

Indicator ID	Final rating							Consensus 'in favour of inclusion' (ratings 5-7)
	1 = Strongly in favour of exclusion	2 = In favour of exclusion	3 = Somewhat in favour of exclusion	4 = Neither in favour of exclusion nor inclusion	5 = Somewhat in favour of inclusion	6 = In favour of inclusion	7 = Strongly in favour of inclusion	
**DBM-STRAT**	0.05	0.11	0.05	0.11	0.21	0.32	0.16	0.68
**DBM-SAFET**	0.21	0.58	0.05	0.00	0.11	0.05	0.00	0.16
**DBM-FARM**	0.00	0.21	0.26	0.11	0.21	0.21	0.00	0.42
**DBM-PARENT**	0.00	0.00	0.00	0.16	0.16	0.37	0.32	**0.84**
**DBM-BREAST**	0.00	0.00	0.05	0.05	0.16	0.37	0.37	**0.89**
**DBM-WORK**	0.00	0.11	0.11	0.00	0.32	0.37	0.11	**0.79**
**DBM-CODE**	0.00	0.00	0.00	0.00	0.21	0.21	0.58	**1.00**
**DBM-COMPL**	0.00	0.00	0.00	0.11	0.00	0.47	0.42	**0.89**
**DBM-DONAT**	0.00	0.05	0.00	0.16	0.21	0.47	0.11	**0.79**
**DBM-INGRED**	0.05	0.21	0.37	0.00	0.11	0.21	0.05	0.37
**DBM-FORTI**	0.05	0.05	0.00	0.11	0.47	0.26	0.05	**0.79**
**DBM-TRAD**	0.00	0.00	0.05	0.16	0.21	0.42	0.16	**0.79**
**DBM-LOGO**	0.05	0.00	0.05	0.26	0.26	0.16	0.21	0.63
**DBM-PROG**	0.26	0.47	0.05	0.11	0.00	0.11	0.00	0.11
**DBM-SIZE**	0.00	0.11	0.16	0.11	0.16	0.26	0.21	0.63
**DBM-ACCESS1**	0.16	0.37	0.21	0.05	0.05	0.16	0.00	0.21
**DBM-ACCESS2**	0.11	0.42	0.21	0.16	0.11	0.00	0.00	0.11

2.7. Third Delphi round: qualitative feedback

Table s7: Qualitative feedback provided during the third Delphi round

Feedback from expert panel	How we addressed the feedback
**1. STRAT1-DBM**: Does the company's commitment to improving population nutrition and health (where it exists) specifically mention undernutrition (wasting, stunting, underweight, micronutrient deficiencies), and/or food insecurity?	
1.1 Definition of food insecurity as lack of nutritious food and not only caloric deficit (message co-opted by the food industry)	We removed the term "food insecurity" to avoid any misinterpretation. Improving the availability of and access to nutritious food is targeted by indicators within the ACCESS domain.
1.2 "Green-washing is possible" here by only mentioning undernutrition/food insecurity, which does not imply ethical conduct (e.g., distributing ultra-processed food might help reduce food insecurity but can contribute to malnutrition/NCDs) → would it be possible for organizations to conduct a risk assessment of the foods they are for example distributing in food provision projects?	This is correct. We removed the term "food insecurity" to avoid any misinterpretation. This is only one of many indicators and we believe it is of relevance if companies publicly commit to address these issues. In addition, the current BIA-Obesity tool already includes a comparable indicator focusing on obesity and NCDs, which will be complemented by the proposed indicator on undernutrition.
1.3 Some efforts to reduce food insecurity can be detrimental for obesity prevention	This indicator is about a company's overall commitment to address undernutrition; specific actions will be assessed as part of the remaining domains of BIA-DBM, assuring that actions addressing undernutrition do not negatively impact other forms of malnutrition.

1.4 In favor of inclusion if there is another indicator related to overweight and obesity and diet-related NCDs	The current BIA-Obesity tool includes such an indicator, which will be complemented by the proposed indicator to address both, under- and overnutrition.
**2. STRAT2-DBM**: Does the company commit to and/or report on ensuring food safety by complying with the Codex Alimentarius General Principles of Food Hygiene?	
2.1 Food safety should be a basic regulated standard, not an optional commitment	*Experts in favour of exclusion. This indicator will not be part of the adapted tool.*
2.2 Not so relevant in the sense that food safety (in general) is quite strongly regulated in comparison to obesity prevention efforts → if included specify which elements it covers	*Experts in favour of exclusion. This indicator will not be part of the adapted tool.*
**3. STRAT3-DBM:** Does the company commit to and/or report on providing opportunities for small-scale farmers to access their supply chains, including fair contracts, training and support, and infrastructure investment?	
3.1 Easily misused and requires rigor in assessment → scoring criteria could factor in the extent to which this is implemented by the company in practice	*Experts in favour of exclusion. This indicator will not be part of the adapted tool.*
3.2 Might be more relevant to know if the companies/food industry have policies/due diligence processes related to the livelihoods and human rights of small-scale farmers (e.g. UNGP BHR)	*Experts in favour of exclusion. This indicator will not be part of the adapted tool.*
3.3 Is important to know if priority is given to safe and nutritious food value chains; however, adding "fresh food"/"nutritious food"/"nutrient dense food" might not solve the problem	*Experts in favour of exclusion. This indicator will not be part of the adapted tool.*
3.4 Difficult from a supply chain perspective because of the multinational context (with centralized supply chains), and different organizational structures (incl. franchising)	*Experts in favour of exclusion. This indicator will not be part of the adapted tool.*
3.5 "Providing opportunities" is key but this might disrupt local markets in communities → may be useful to add "promote and support environmentally friendly practices that maintain local ecosystems and resources and markets"	*Experts in favour of exclusion. This indicator will not be part of the adapted tool.*
3.6 Not directly related to food security and the DBM → suggestion to focus on more relevant indicators	*Experts in favour of exclusion. This indicator will not be part of the adapted tool.*
3.7 Only makes senses adding this indicator if the tool also covers other 'social indicators' - either cover them fully or not at all	*Experts in favour of exclusion. This indicator will not be part of the adapted tool.*

**4. STRAT4.1-DBM:** Does the company commit to and/or report on providing all employees at least 14 weeks of paid maternity leave?	
4.1 As an employee-specific indicator might be less important, depending on the number of workers in each sector/department within the company → potentially add context by saying 'it may be more or less relevant to include this indicator' and don't give elevated scores when these are likely to have little impact	We consider scoring and/or weighting all workplace indicators lower compared to other indicators/domains but do believe it is important that these aspects are represented in the assessment tool to create innovation and transformation pathways.
4.2 Clarification of the term "all employees" to avoid ambiguity → use "primary caregiver" instead	Thank you for this suggestion, we rephrased the indicator accordingly.
4.3 If split from breastfeeding indicator, suggest to change to parental leave again; this also supports child care for siblings and apart from nutrition prevents child labour and children not attending school	Thank you for this suggestion, we rephrased the indicator accordingly.
4.4 At least 14 weeks is in line with much literature, however WHO recommends 6 months exclusive breastfeeding	We suggest starting with 14 weeks to aim for realistic implementation, however all indicators should be revised / adjusted over time to reflect their appropriateness.
**5. STRAT4.2-DBM:** Does the company commit to and/or report on providing breastfeeding mothers with appropriate working conditions (e.g., offering flexible working arrangements), and facilities at work (e.g., to breastfeed, express and store breastmilk)?	
5.1 Organizations can set up breastfeeding facilities, regardless of whether they use labor brokers	This is correct. Informal labor could also benefit from such facilities.
5.2 "breastfeeding parents" might be more gender-inclusive	Thank you for this suggestion. For now, we decided to continue with the proposed wording. However, all indicators should be revised / adjusted over time to reflect their appropriateness.
5.3 Weigh these workplace wellness indicators really low (like 5% as ATNI does), or even only 1%	Thank you for this suggestion, which we will consider at the next stage of our adaptation process.
5.4 'Facilities' and 'working conditions' could potentially be split, or have different scores (higher if both, lower if only one)	Thank you for this suggestion, which we will consider at the next stage of our adaptation process.
**6. STRAT5-DBM:** Does the company commit to and/or report on having measures in place to ensure that employees can practice a healthy diet at an affordable price during working hours (e.g., company cafeterias serving nutritious foods, food vouchers which can be used for nutritious foods)?	
6.1 Consider not applying to QSRs?	Some QSRs have relatively more nutritious food options as well (e.g., chicken wraps, salads, etc.). We believe that companies should be encouraged to offer these (usually more expensive) options to their employees during working hours (instead of potentially only offering the cheapest and less nutritious menu).

6.2 Minor issue and could be easily manipulated	We consider scoring and/or weighting all workplace indicators lower compared to other indicators/domains but do believe it is important that this aspect is represented in the assessment tool to create innovation and transformation pathways.
6.3 Measuring this indicator is challenging, However, some measurements could be relatively easy assessed (e.g cafeteria prices, placement of food, options of safe nutritious food on the menu, etc)	Thank you for this suggestion, which we will consider at the next stage of our adaptation process (= scoring criteria).
6.4 Weigh these workplace wellness indicators really low (like 5% as ATNI does), or even only 1%	Thank you for this suggestion, which we will consider at the next stage of our adaptation process.
**7. STRAT6-DBM:** Does the company commit to and/or report on complying with 'The International Code of Marketing of Breastmilk Substitutes' and all subsequent WHA resolutions, as well as the Codex Alimentarius 'Code of Hygienic Practice for Powdered Formulae for Infants and Young Children'?	
7.1 These are two different things - the first part of the indicator should have its own indicator	Thank you for this comment based on which we removed the previous reference to CAC/RCP 66 - 2008 (Codex Alimentarius). Instead, we added the perspective of nutritional composition of infant formula by referring to CXS 72-1981: Standard for Infant Formula and Formulas for Special Medical Purposes Intended for Infants (here)
**8. STRAT7-DBM:** Does the company commit to and/or report on producing, marketing, and distributing commercially available complementary foods only in line with national evidence-informed recommendations, or (if not available) international guidance (e.g. the International Code of Marketing of Breastmilk Substitutes, Codex Alimentarius, or WHO NPPM)?	
8.1 Risk that companies use “loopholes” in regulations to continue producing unhealthy products but with health or nutrition claims (e.g. high in vitamins, fiber, etc) → depends on comprehensiveness of national regulations	Thank you for this comment, which is an important aspect to be considered. So far, both The Code and Codex Alimentarius standards offer guidance on nutritional labeling of commercially available complementary foods, including the use of nutrition and health claims.
**9. STRAT8-DBM:** Does the company commit to and/or report on applying discounts on nutritious food that is considered safe (i.e. within "use-by-date" and unspoiled) but that cannot be sold at the regular price, and/or donating it to people in need?	
9.1 No packaged or ultra-processed products should be considered nutritious. Very clear criteria should be provided what is considered nutritious; on the other hand, there is some minimally processed food which is essential to food back users (e.g. pasta, beans, tinned vegetables) → maybe rather use stipulation around UPFs/NOVA classification or HFSS rather than packaging?	We elaborated a definition on what foods / products would be considered ‘nutritious’ (see 18.2). Per definition, this usually excludes UPFs and HFSS foods. We believe that also packaged, minimally processed foods, such as beans and tinned vegetables could be considered nutritious and therefore would not limit this indicator to non-packaged foods.

9.2 From a CSR perspective reflects well on the company but it does not solve the problem of food insecurity (and is also not a dignified way to access foods). The company should be really committed doing this for a large percentage of their product portfolio and not just sometimes for some random products → could be worked into scoring criteria	Thank you for this comment. Indeed, food insecurity cannot be solved through such measures. Still, actions like these can represent one building block of ensuring relatively short-term food security. We consider taking the comparatively lower relevance of this indicator into account in the scoring.
9.3 “Ugly fruits and vegetables” should be excluded or separated from this indicator (something different)	Ugly fruits and vegetables are nutritious and if they are considered safe but cannot be sold at the regular price, and/or are donated to people in need, this would/should also be covered by this indicator.
9.4 Rather a sustainability/food waste issue (than a malnutrition-related issue) → what quantities need to be provided to what proportion of the population to have a public health impact?	Thank you for this comment. Indeed, this indicator targets both food waste and food accessibility. Still, actions like these can represent one building block of ensuring relatively short-term food security. We consider taking the comparatively lower relevance of this indicator into account in the scoring.
9.5 Difficult to measure	Thank you for this suggestion, which we will consider at the next stage of our adaptation process.
9.6 Unsure about purpose of this indicator - there are already other indicators on healthy foods being cheaper than unhealthy options	The proposed indicator is not based on affordability, but rather accessibility aspects. It targets nutritious foods close to the expiry date, aiming to make these foods available to priority populations and to avoid food waste. It is correct that there is an existing ACCESS indicator that encourages companies to make nutritious foods overall more affordable compared to less nutritious foods.
9.7 Don’t think the phrase “cannot be sold at the regular price” is necessary.	We added this phrase to differentiate it from an existing ACCESS indicator that encourages companies to make nutritious foods more affordable compared to less nutritious foods. The proposed indicator targets nutritious foods close to the expiry date, to make these foods available to priority populations and to avoid food waste.
**10. FORM1-DBM:** What commitments or actions has the company taken with respect to increasing content of fruits, vegetables, legumes, nuts and/or wholegrains in their food products? Does the company routinely report on this measure across its product portfolio and progress against relevant commitments and best practice standards?	
10.1 Rephrasing or rewording to make it clear that this would only apply to foods that meet a general threshold of healthiness → - e.g., instead of "...in their food products" could be "...in their healthier food products	*Experts in favour of exclusion. This indicator will not be part of the adapted tool.*
10.2 Instead of ‘content’, use ‘proportion’	*Experts in favour of exclusion. This indicator will not be part of the adapted tool.*

10.3 Suggest the following rephrasing: 'What commitments or actions has the company taken to increase the content of minimally processed fruits, vegetables, legumes, nuts, and/or whole grains in their food products? Does the company routinely report on this measure across its product portfolio and progress against relevant commitments and best practice standards, including efforts to avoid ultra-processing that reduces the fibre and nutrient profile of the whole ingredients?'	*Experts in favour of exclusion. This indicator will not be part of the adapted tool.*
**11. FORM2-DBM:** For companies producing/using/distributing fortified or enriched foods, does the company commit to and/or report on complying with national, regional, or international standards or recommendations on food fortification?	
11.1 Indicator too vague. Should focus on staple grains and staple condiments (oil and salt)	Thank you for this suggestion, we rephrased the indicator accordingly. We still suggest that the actual selection of foods to be fortified/enriched should be based on particularly national and regional standards and recommendations to choose the most effective medium.
11.2 Consider an explicit notion of asking companies to commit NOT to fortify/enrich products that are unhealthy/less healthy	Thank you for this suggestion. This would imply excluding various staple foods and condiments (e.g., oils, salt, soy sauce) from being fortified/enriched. We suggest that the actual selection of foods to be fortified/enriched should be based on particularly national and regional standards and recommendations to choose the most effective medium.
11.3 Add the word "applicable" to → "complying with applicable national, regional..." to avoid that they follow just one random guidelines rather than the most relevant	Thank you for this suggestion, we rephrased the indicator accordingly.
11.4 Better to include only "national" standards (fortification levels might differ significantly between countries)	Thank you for this suggestion. We rephrased the indicator accordingly. However, where no national standards are available, both regional and international standards (particularly the first) still provide relevant and useful recommendations.
**12. FORM3-DBM:** Does the company commit to and/or report on increasing the use of traditional, culturally acceptable, and nutritious foods (e.g. sweet potato, amaranth, millets) in food production, in line with national or international dietary guidelines?	
12.1 The indicator should mention code safety guidelines (aflatoxins should be considered)	The BIA-Obesity assessment looks at food industry actors with the biggest market shares in the country. These are often multinational companies with usually high food-safety and hygiene standards. We therefore consider the proposed addition not necessary.

12.2 Food companies could use these ingredients to produce processed / ultra-processed products → indicator should be restricted to healthy foods only	Thank you for this suggestion, we rephrased the indicator accordingly.
12.3 Concern about applicability of this indicator to multinational companies. On the other side, might be a way to encourage national companies to invest in this area	Thank you for this comment. Indeed, this aspect may create innovation and transformation pathways.
**13. LABEL1-DBM:** Does the company commit to and/or report on only using an official (government-endorsed) fortification logo (where available) to label fortified products?	
13.1 Logo might be used as a marketing strategy, and in some settings preferably not used as fortification has bad reputation among consumers	*Experts in favour of exclusion. This indicator will not be part of the adapted tool.*
13.2 Issue already covered by FORM2-DBM, additional burden on participants	*Experts in favour of exclusion. This indicator will not be part of the adapted tool.*
13.3 Indicator should be limited to staple grains and condiments (as indicator FORM2-DBM)	*Experts in favour of exclusion. This indicator will not be part of the adapted tool.*
13.4 As opposed to what other logo? Should be accompanied by consumer education, otherwise logo just useful for M&E purposes	*Experts in favour of exclusion. This indicator will not be part of the adapted tool.*
13.5 Also depends on monitoring at country level; does using gov logo ensure bioavailability and safety? - it not monitored may be misleading	*Experts in favour of exclusion. This indicator will not be part of the adapted tool.*
**14. PROMO1-DBM:** For nutrition programs supported or conducted by the company, does the company commit to and/or report that all programs are evidence-based and aligned with relevant national or international guidelines, and exclude product or brand level branding? (max. scoring if the company is not supporting / conducting nutrition programs)	
14.1 1 Clarify the type of nutrition programs: external (funding by CSI/CSR initiatives etc) or internal? → e.g. make clear that nutrition programmes for schools should be included here	*Experts in favour of exclusion. This indicator will not be part of the adapted tool.*
**15. ACCESS1-DBM:** Does the company commit to and/or report on offering healthy foods in packaging / portion sizes that suit a limited budget to increase the accessibility of these foods for low-income populations?	
15.1 Clarify if it only refers to "commercially available food products" or if it also includes fresh food (fruits, vegetables etc). → important to clarify	*Experts in favour of exclusion. This indicator will not be part of the adapted tool.*
15.2 Clarify the word “healthy”. Packaged foods are usually processed or ultra-processed → strict criteria needed what products ought to be included here	*Experts in favour of exclusion. This indicator will not be part of the adapted tool.*
15.3 May be difficult to measure	*Experts in favour of exclusion. This indicator will not be part of the adapted tool.*

15.4 Can reduce safety risk and increase monitoring, e.g., of food fortification if not "decanted" or "repackaged" products (e.g., oil, rice etc.) but it can also drive up prices and increase plastic waste	*Experts in favour of exclusion. This indicator will not be part of the adapted tool.*
**16. ACCESS2-DBM:** Does the company commit to and/or report on improving the availability, affordability and accessibility of healthy foods relative to unhealthy foods for population groups experiencing or at high risk of undernutrition and micronutrient deficiencies?	
16.1 Many interpretation issues and risk that one off pilots are being framed as corporate commitments	*Experts in favour of exclusion. This indicator will not be part of the adapted tool.*
**17. ACCESS3-DBM:** Does the company commit to and/or report on improving the availability, affordability and accessibility of fortified foods (e.g. dietary oils, salt, flour), in line with national, regional, or international standards or recommendations on food fortification?	
17.1 Keep it for retailers only and rephrase → instead of saying "improving", maybe reword to focus on "equitable" distribution (to ensure that those products are also offered in rural or low income areas)	*Experts in favour of exclusion. This indicator will not be part of the adapted tool.*
17.2 Voluntary fortification effort by the industry might help in the short term to transition to mandatory fortification in the medium term (the goal)	*Experts in favour of exclusion. This indicator will not be part of the adapted tool.*
**18. Further comments and suggestions**	
18.1 The first indicator should consider the DBM by including overweight, obesity and diet-related NCDs in addition to undernutrition and food insecurity	The current BIA-Obesity tool already includes such an indicator, which will be complemented by our proposed first indicator to address both, under- and overnutrition.
18.2 Provide/establish definitions of healthy, unhealthy, nutritious, etc. based on recommendations, guidelines, literature, etc.	Thank you for this important suggestion. We consider using the following definitions:<br>• Healthy diets: Diets that are in line with applicable national, regional or (if not available) international food-based dietary guidelines (FAO). Usually, healthy diets are adequate (providing enough essential nutrients to prevent deficiencies and promote health, without excess), balanced (in energy intake, and energy sources (i.e., fats, carbohydrates, proteins)), moderate (in consumption of foods, nutrients or other compounds associated with detrimental health effects), and diverse (including a variety of nutritious foods within and across food groups to favour nutrient adequacy and consumption of other health promoting substances) (FAO/WHO)

	• Nutritious foods: Safe foods that contribute essential nutrients such as vitamins and minerals (micronutrients), fibre and other components to healthy diets that are beneficial for growth, health and development, guarding against malnutrition. In nutritious foods, the presence of nutrients of public health concern is minimized (FAO)
18.3 Indicator(s) on the food environment is missing (e.g., responsible marketing/ not marketing unhealthy/ less healthy food (groups) to children)	These issues are already covered by the current BIA-Obesity tool, as food marketing also heavily affects the overweight/obesity pandemic.
18.4 Many of the discussed practices could be considered 'health washing" but this is unavoidable, and doesn't necessarily rule out an indicator as being useful. Still relevant from an accountability perspective. So don't worry too much about this criticism	Thank you for this valuable comment and perspective.
18.5 When the indicator refers to commercially manufactured products, it should be clearly stated as 'food products', whereas when it refers to whole food, it should be categorized as 'food groups' → fruits e.g. would be a food group	This is an important comment. In line with the existing BIA-Obesity tool, we use "foods" as a generic term for both food products and food groups, but will use the more specific terms where appropriate.
18.6 Employee-related indicators could be removed in country contexts if the workforce contributing to food manufacturing/retailing is relatively low → encourage BIA-DBM users to reflect on the utility of these indicators in their context	Thank you for this suggestion, which we will consider at the next stage of our adaptation process.
**19. Evaluation (Comments)**	
19.1 Well-organized process, informative, effective and easy to follow	
19.2 Study with significant potential	
19.3 Looking forward to seeing final results, the indicators and the next steps	
19.4 Particularly workshops were useful to hear from experts and discuss nuances of indicators	

4. Differences between protocol and manuscript

This study is based on a protocol developed and prospectively registered and published online through the Open Science Framework (registration DOI: 10.17605/OSF.IO/CJE4T) before data were collected (19). In the following, we describe differences between the protocol and the manuscript:

- Instead of following the *Guidance on Conducting and Reporting Delphi Studies* (CREDES) (20), we followed the recently published *Delphi studies in social and health sciences—Recommendations for an interdisciplinary standardized reporting* (DELPHISTAR) guidance (21), as the latter was specifically designed to be applied in social and health sciences research.

- We did not conduct inductive content analyses of the qualitative feedback received during the three Delphi rounds. Instead, for each indicator and Delphi round, we summarized the main discussion points and how we addressed them, and presented this overview in Tables s4, s5, and s7.

5. Reflexivity statement

In line with best practice recommendations for qualitative research (22, 23), the first author (CK) and the last author (PvP) jointly reflect in the following on how their backgrounds, experiences, and values may have influenced the research process and interpretation of findings. CK is a PhD student in Epidemiology and Public Health, with academic training in Business (BA, with a focus on tourism), Nutritional Science (BSc), and Public Health (MSc). PvP is a medical doctor and professor of public health nutrition and holds master's degrees in Global Politics (MSc) and Political Science, Economics and Law (MA). Both CK and PvP are guided by a belief in the principles of free market economies, while also recognizing the necessity of government intervention in instances where market failure may occur or where human, animal, or environment health is at risk. Within the context of this study, CK and PvP approached the research from an external standpoint, as neither has previously worked in or for the food industry sectors under examination. They share the view that achieving sustainable human and planetary health will require substantial shifts in global consumption patterns, including a marked reduction in animal-based products and ultra-processed foods. At the same time, they acknowledge the critical role the food industry has played in reducing undernutrition and food insecurity across many regions of the world during the past decades. As such, they consider a food system without a major role of the private food industry (e.g. through a return to full self-sufficiency in food production) to be both unrealistic and undesirable from a global health perspective.

6. Delphi studies in social and health sciences—Recommendations for an interdisciplinary standardized reporting (DELPHISTAR) guidance (21)

Table s8: DELPHISTAR guidance

Topic	Section	Item	Checklist Item	Location where item is reported	Exemplary answer
I Title and Abstract		1	Identification as a Delphi procedure in the title	Page 1	What is a public health intervention? Results of a Delphi study.
		2	Identification as a Delphi procedure in the abstract	Page 3	A Delphi procedure was selected to answer the research question.
		3	Structured abstract	Pages 3-4	e.g., background, method, results and discussion
II Context	**Formal**	4	Information about the sources of funding	Page 30	The Delphi study was funded by [SOURCE].
		5	Information about the team of authors and/or researchers (e.g., discipline, institution)	Page 10	The Delphi study was conducted by an interdisciplinary team with representatives from medicine, public health, and health promotion.
		6	Information about method consulting	Suppl material: Page 8	The study group was advised by external experts from [INSTITUTION] regarding statistics.<br>Or:<br>No outside consulting in regard to method took place.
		7	Information about the project background	Page 30	The Delphi survey was part of a mixed-methods study on [AIM].
		8	Information about the study protocol	Page 8	The study protocol is available at [LINK].
	**Content**	9	Justification of the chosen method (Delphi procedure) to answer the research question	Page 8-9	The Delphi method is suitable for answering the research question because it systematically gathers the judgments of different expert groups and can identity agreement and disagreement.
		10	Aim of the Delphi procedure (e.g., consensus, forecasting)	Page 8	The aim of the Delphi study is to find consensus on criteria to define a public health intervention.
III Method	**Body & Integration of knowledge**	11	Identification and elucidation of relevant expertise, spheres of experience, and perspectives (e.g., theory, practice, affected groups, disciplines)	Page 9; Suppl material: Pages 2-3	The experts should represent the sciences and clinical practice because [REASON].

Topic	Section	Item	Checklist Item	Location where item is reported	Exemplary answer
		12	Handling of knowledge, expertise and perspectives which are missing or have been deliberately not integrated	Page 24-25	If it is not possible to recruit experts specialized in [AREA], this is openly communicated to the other respondents during the Delphi process.
		13	Basic definition of expert[1]	Page 9; Suppl material: Pages 2-3	A person who has been active in the area for at least [NUMBER] years is considered to be an expert.
	**Delphi variations**	14	Identification of the type of Delphi procedure and potential modifications (e.g., classic Delphi, real-time Delphi, group Delphi)	Page 8	A classic Delphi procedure was used [LITERATURE REFERENCE].
		15	Justification of the Delphi variation and modifications, including during the Delphi process, if applicable	Page 8-9	If the willingness to participate clearly decreases between the first and second rounds, a third round will not be held.
	**Sample of experts**	16	Selection criteria for the experts (per round if there are different expert groups)	Pages 9; Suppl material: Pages 2-3	All of the experts who met the definition were invited to the first round.<br>All of the experts who completed the previous round were invited to participate in the subsequent round.
		17	Identification of the experts	Pages 9; Suppl material: Pages 2-3	The experts were identified based on publications in [DATABASE].
		18	Information about recruiting and any subsequent recruiting of experts	Suppl material: Pages 2-3	The experts were informed about the Delphi study and invited to participate.
	**Survey**	19	Elucidation of the content development for the questionnaire[2]	Pages 10-11; Suppl material: Table s1	The questionnaire was developed based on the results of systematic reviews [LITERATURE REFERENCE].
		20	Description of the questionnaire (content and structure)	Pages 12-14	The questionnaire was divided into three segments on [TOPICS]. The statements made in the questionnaire were evaluated using standardized items, with the option to comment in free-text boxes.
	**Delphi rounds**	21	Number of Delphi rounds	Page 10, Figure 1	Three Delphi rounds were held.
		22	Information about the aims of the individual Delphi rounds	Pages 12-14	The first Delphi round focused on exploring relevant aspects. These aspects were then presented to the experts in the second Delphi round for standardized evaluation.
		23	Disclosure and justification of the criterion for discontinuation	Page 10	The number of rounds was defined in advance to be a maximum of three rounds.

Topic	Section	Item	Checklist Item	Location where item is reported	Exemplary answer
	Feedback	24	Information about what data was reported back per round	Pages 12-14	In terms of feedback, we shared the statistical results plus the summary of the open responses.
		25	Information on how the results of the previous Delphi round were fed back to the experts surveyed (e.g., via frequencies, mean values, measures of dispersion, listing of comments)	Pages 12-14	Mean values, standard deviations and percentage frequency distributions were reported.
		26	Information on whether feedback was differentiated by specific groups (e.g., by field of expertise, institutional affiliation)	Page 12	The feedback was aggregated across all expert groups.
		27	Information about how dissent and unclear results were handled	Pages 12-14	The results showing dissent were presented again for evaluation in the next Delphi round.
	Data analysis	28	Disclosure of the quantitative and qualitative analytical strategy	Pages 12-14	The quantitative items were descriptively analyzed. The open-ended items were analyzed using thematic analysis [LITERATURE REFERENCE].
		29	Definition and measurement of consensus	Page 13-14	Consensus was defined as percentage agreement, meaning that agreement was assumed if at least 80% of the respondents agreed on an item.
		30	Information on group-specific analysis or weighting of experts (e.g., theory vs. practice, discipline-specific analysis)	Page 12	In the analysis, the mean values for percent agreement are weighted for each expert group in terms of the number of group members.
IV Results	Delphi process	31	Illustration of the Delphi process (e.g., in a flow chart)	Figure 1	A summary of the process is illustrated in a flow chart (Figure 1).
		32	Information about special aspects during the Delphi process (e.g., deviations from the intended approach with justification)	N/A	During the Delphi procedures the political discussion mentioned climate change and the effects on health. It is possible that this influenced the experts' responses.
		33	Number of experts per round (both invited and participating)	Page 15, Table 1	The number of experts participating in the first Delphi round was [NUMBER], and the number of experts in the second round was [NUMBER]. This corresponds to a response rate of [NUMBER]% in the first round and [NUMBER]% in the second round.

Topic	Section	Item	Checklist Item	Location where item is reported	Exemplary answer
	**Results**	34	Presentation of the results for each Delphi round and the final results	Pages 15-18, Figure 2; Suppl material: Table s3-s7, Figure s3	In the first Delphi round [NUMBER]% of the respondents agreed, in the second [NUMBER]%, and in the third [NUMBER]%.
V Discussion	**Quality of findings**	35	Highlighting the findings from the Delphi study	Pages 21-24, Table 2, Figure 3	The central findings can be summarized as follows: [STATE FINDINGS].
		36	Validity of the results (e.g., transferability of the findings)	Pages 24-26	The results are not transferable to other countries due to different legal regulations.
		37	Reliability of the results (e.g., split half, inter-rater reliability)	Page 12	The responses in the free-text comments were analyzed by two independent reviewers [SPECIFY].
		38	Reflection on potential limitations (e.g., distortion, skewing, bias)	Pages 24-25	The results are to be viewed critically with regard to the composition of the panel because [REASONS].

7. References

1. Gilmore AB, Fabbri A, Baum F, Bertscher A, Bondy K, Chang H-J, et al. Defining and conceptualising the commercial determinants of health. The Lancet. 2023;401(10383):1194-213.
2. Klinger C, Alaba O, Delobelle P, Holliday N, Lambert EV, Leibinger A, et al. Recommendations for and best practices by the formal food industry to address undernutrition and the double burden of malnutrition: a scoping review protocol. Open Science Framework. 2023.
3. Beiderbeck D, Frevel N, von der Gracht HA, Schmidt SL, Schweitzer VM. Preparing, conducting, and analyzing Delphi surveys: Cross-disciplinary practices, new directions, and advancements. MethodsX. 2021;8:101401.
4. Niederberger M, Renn O. Das Gruppendelphi-Verfahren. Wiesbaden: Springer VS; 2018.
5. Sacks G, Vanderlee L, Robinson E, Vandevijvere S, Cameron AJ, Ni Mhurchu C, et al. BIA-Obesity (Business Impact Assessment-Obesity and population-level nutrition): A tool and process to assess food company policies and commitments related to obesity prevention and population nutrition at the national level. Obes Rev. 2019;20 Suppl 2:78-89.
6. Förster B, von der Gracht H. Assessing Delphi panel composition for strategic foresight — A comparison of panels based on company-internal and external participants. Technological Forecasting and Social Change. 2014;84:215-29.
7. Coste M, Pereira L, Charman A, Petersen L, Hawkes C. 'Hampers' as an effective strategy to shift towards sustainable diets in South African low-income communities. Development Southern Africa. 2023;40(2):350-72.
8. Laurie SM, Faber M, Claasen N. Incorporating orange-fleshed sweet potato into the food system as a strategy for improved nutrition: The context of South Africa. Food Research International. 2018;104:77-85.
9. Chevrollier N, Bults R, Sprenger T, Danse M, Poniatowski B, O'Neill K. Access to Food and Improved Nutrition at the Base of the Pyramid: Five business interventions to achieve social impact, financial sustainability and scale. 2012.
10. Wimalawansa SJ. Rational Food Fortification Programs to Alleviate Micronutrient Deficiencies. J Food Process Technol. 2013;4:257.
11. FAO. The state of food and agriculture 2013. Rome: Food and Agriculture Organization of the United Nations; 2013.
12. Mabaya E, Jordaan D, Malope P, Monkhei M, Jackson J. Attribute preferences and willingness to pay for fortified cereal foods in Botswana. Agrekon. 2010;49(4):459-83.
13. FAO, ILSI. Preventing micronutrient malnutrition: A guide to food-based approaches - A manual for policy makers and programme planners Washington D.C., USA1997 [Available from: https://www.fao.org/4/x5244e/X5244e00.htm#TopOfPage.
14. Mason J, Mannar V, Mock N. Controlling Micronutrient Deficiencies in Asia. Asian Development Review. 1999;17(01n02):66-95.
15. Lutter CK. Macrolevel approaches to improve the availability of complementary foods. Food Nutr Bull. 2003;24(1):83-103.
16. Hystra. Marketing nutrition for the base of the pyramid: Introducing successful practices for improved access to nutritious complementary foods: Key lessons from case studies. GAIN; 2014.
17. van Liere MJ, Tarlton D, Menon R, Yellamanda M, Reerink I. Harnessing private sector expertise to improve complementary feeding within a regulatory framework: Where is the evidence? Matern Child Nutr. 2017;13 Suppl 2(Suppl 2).

18. Mannar MG, van Ameringen M. Role of public-private partnership in micronutrient food fortification. Food Nutr Bull. 2003;24(4 Suppl):S151-4.
19. Klinger C, Rehfuess EA, Delobelle P, Theurich MA, Holliday N, Okanmelu EC, et al. Adaptation of the INFORMAS BIA-Obesity framework for countries facing a double burden of malnutrition: a Delphi study protocol. Open Science Framework. 2024.
20. Jünger S, Payne SA, Brine J, Radbruch L, Brearley SG. Guidance on Conducting and REporting DElphi Studies (CREDES) in palliative care: Recommendations based on a methodological systematic review. Palliat Med. 2017;31(8):684-706.
21. Niederberger M, Schifano J, Deckert S, Hirt J, Homberg A, Köberich S, et al. Delphi studies in social and health sciences—Recommendations for an interdisciplinary standardized reporting (DELPHISTAR). Results of a Delphi study. PLOS ONE. 2024;19(8):e0304651.
22. O'Brien BC, Harris IB, Beckman TJ, Reed DA, Cook DA. Standards for reporting qualitative research: a synthesis of recommendations. Acad Med. 2014;89(9):1245-51.
23. Tong A, Sainsbury P, Craig J. Consolidated criteria for reporting qualitative research (COREQ): a 32-item checklist for interviews and focus groups. International Journal for Quality in Health Care. 2007;19(6):349-57.
